# Single-cell chromatin state transitions during epigenetic memory formation

**DOI:** 10.1126/sciadv.aeb0060

**Published:** 2026-07-23

**Authors:** Taihei Fujimori, Abby R. Thurm, Simon Gaudin, Carolina Rios-Martinez, Benjamin R. Doughty, Michaela M. Hinks, Joydeb Sinha, Derek Le, Antonina Hafner, William J. Greenleaf, Alistair N. Boettiger, Lacramioara Bintu

**Affiliations:** ^1^ Department of Bioengineering, Stanford University, Stanford, CA, USA.; ^2^ Chan Zuckerberg Biohub, San Francisco, CA, USA.; ^3^ Biophysics Program, Stanford University, Stanford, CA, USA.; ^4^ Department of Genetics, Stanford University, Stanford, CA, USA.; ^5^ Department of Chemical & Systems Biology, Stanford University, Stanford, CA, USA.; ^6^ Department of Dermatology, Program in Epithelial Biology, Stanford University, Stanford, CA, USA.; ^7^ Program in Cancer Biology, Stanford University, Stanford, CA, USA.; ^8^ Department of Developmental Biology, Stanford University, Stanford, CA, USA.; ^9^ Department of Applied Physics, Stanford University, Stanford, CA, USA.

## Abstract

Repressive chromatin modifications compact chromatin and mediate heritable gene silencing, but how structural changes quantitatively relate to epigenetic memory remains unclear. Using targeted recruitment of the KRAB repressor to induce H3K9me3 at a reporter gene, combined with single-molecule 3D chromatin imaging, we show that irreversible silencing is associated with large-scale chromatin compaction across tens of kilobases. In contrast, histone deacetylation produces reversible silencing without such compaction. Despite substantial single-cell heterogeneity, average compaction at the end of silencing quantitatively predicts epigenetic memory weeks after KRAB removal. Here, memory arises not through stable H3K9me3 domains but rather through a dynamic handoff in which H3K9me3 is gradually lost and replaced by DNA methylation. Stochastic simulations recapitulating these dynamics suggest that compaction enhances read-write feedback to promote this transition. Similar compaction is observed at endogenous loci during differentiation and fate commitment, suggesting that spatial organization may be predictive of epigenetic memory in other systems.

## Teaser

Large-scale chromatin compaction quantitatively predicts the percentage of cells with durable epigenetic memory of gene silencing.

## INTRODUCTION

Chromatin modifications on both DNA and histone tails are fundamental to eukaryotic gene regulation, and controlling diverse biological processes such as differentiation (*1*), immune responses (*2*), metabolic stress responses (*3*), or neural wiring (*4*), and their misregulation often results in disease (*5*). Chromatin-mediated gene control is tightly linked to three-dimensional (3D) chromatin organization. In particular, repressive chromatin modifications such as histone H3 lysine 9 trimethylation (H3K9me3) are associated with 3D compaction of the chromatin polymer (*6*) and lamina association at the nuclear periphery (*7*). Targeted recruitment of the KRAB domain from zinc finger 10 to a gene causes efficient silencing (*8*, *9*) through its direct interaction with KAP1, a corepressor that in turn recruits histone methyltransferase SETDB1 (H3K9me3 writer), histone deacetylases, and heterochromatin protein 1 (HP1, H3K9me3 reader) (*10*, *11*). Both KRAB and HP1 recruitment at a genomic locus induce de novo accumulation of H3K9me3, increase chromatin density (*12*), and reduce chromatin accessibility (*13*). The recruitment of dCas9-KRAB [known as CRISPRi (*9*)] to many loci induces chromatin compaction (*14*), exemplifying another link between repressive chromatin modifications and changes in 3D chromatin organization.

One remarkable feature of heterochromatin-associated modifications is that they are often transmitted across multiple cell divisions to maintain gene expression programs that support cellular identity, called epigenetic memory (*15*). Epigenetic inheritance is achieved through positive feedback mediated by reader-writer modules (*16*). For example, it has been shown that mammalian H3K9 methyltransferase SUV39H1 binds to H3K9me3 through its chromodomain and deposits H3K9me3 onto nearby unmodified nucleosomes (*17*). A similar mechanism has been found for H3K27me3, mediated by the polycomb repressor complex (*18*) and DNA methylation maintained by DNA methyltransferase DNMT1 (*19*). These mechanisms are thought to facilitate spreading of chromatin modifications and maintain the domain of chromatin modifications through cell cycles.

However, often epigenetic memory is not maintained in all cells in the population, even when cells are genetically identical and have been exposed to the same stimulus: Only a fraction of cells will maintain silencing, as evidenced from early experiments with position-effect variegation (*20*), heterochromatin maintenance in yeast (*21*), and after direct gene silencing by recruitment of chromatin regulators at target genes (*13*, *22*, *23*). Intriguingly, the fraction of cells with memory increases with the duration of the signal, for example, the recruitment duration of the chromatin regulator at the target gene (*22*, *24*). Despite the detailed molecular mechanisms proposed for epigenetic gene silencing and inheritance, it is still not understood why epigenetic memory is maintained in a fraction of cells in the population and not others, nor how the information is transferred from recruitment duration to fractional epigenetic memory.

Here, we combined targeted recruitment and release of repressors—which allows us to tune the fraction of cells with epigenetic memory (*22*)—with systematic measurements of histone modifications (*25*) and single-cell chromatin tracing (*26*) to quantitatively connect chromatin state changes to epigenetic memory formation. We found that KRAB recruitment induces stochastic long-term gene silencing epigenetic memory and leads to H3K9me3 accumulation and chromatin compaction across tens of kilobases. This chromatin compaction induced by KRAB recruitment is retained in stably silenced cells after KRAB release despite the fact that H3K9me3 gradually decreases and DNA methylation slowly accumulates after release. By varying the duration of KRAB recruitment and using KRAB mutants with partial loss of function, we generated cell populations with different fractions of stably silenced cells and found that the degree of chromatin compaction across 10 to 20 kb together with H3K9me3 abundance at the end of recruitment can quantitatively predict epigenetic memory weeks later. DNA methyltransferase DNMT3B recruitment was sufficient to induce chromatin compaction and epigenetic memory. In contrast, silencing by short recruitment of histone deacetylase HDAC4 does not lead to epigenetic memory nor large-scale compaction, suggesting that transcriptional silencing is not sufficient to induce chromatin compaction at tens of kilobases scale. By incorporating our measurements into a stochastic simulation of H3K9me3 spreading across a nucleosome array, we show that a H3K9me3 positive feedback model recapitulates slow H3K9me3 decay and epigenetic memory formation after KRAB release. Last, we demonstrated that compaction at the *Nanog* locus is a good predictor of irreversible fate commitment during mouse embryonic stem cell (mESC) differentiation and identified a set of genes exhibiting rapid silencing, slow compaction dynamics during differentiation, mirroring the process observed in our synthetic system.

## RESULTS

### KRAB recruitment causes heritable chromatin compaction

**Fig. 1.**
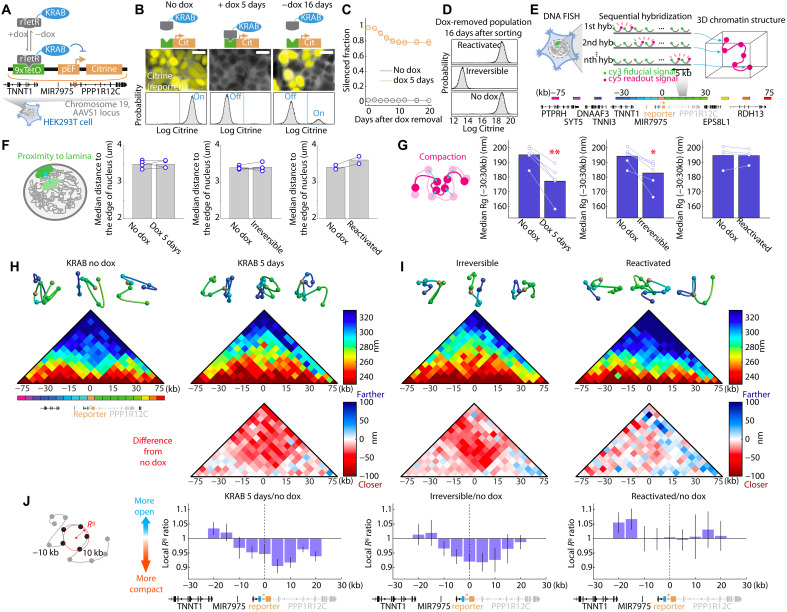
Targeted KRAB recruitment results in 3D chromatin compaction that is retained in irreversibly silenced cells. (**A**) Schematic representation of the reporter system with targeted KRAB recruitment. (**B**) Top: Schematics describing experimental setup and associated fluorescent images. Scale bars, 20 μm. Bottom: Probability density distributions for Citrine fluorescence: number of cells within a particular fluorescence bin as measured by flow cytometry normalized by total number of cells (for the A6 clone, see fig. S1). (**C**) Time course showing the Citrine-silenced fraction [OFF population in (B)] during KRAB release. Lines show the average of three replicates. (**D**) Probability distributions of Citrine expression levels in the irreversibly silenced or reactivated cells following cell sorting (B9 clone). (**E**) Schematic overview of the ORCA technique (top). Genome segments that are imaged are annotated with colored rectangles (bottom). (**F**) Median distance between the DNA FISH spot and the edge of the nucleus. (**G**) Median radius of gyration for the −30- to 30-kb region around the reporter. *P < 0.05 and **P < 0.01 by two-sided paired Student’s *t* test compared to no dox. (**H**) Top: Representative 3D chromatin structure (from −30 to 30 kb). Color code follows the schematic in (E). Middle: Heatmaps of median distances between pairwise detected segments. Bottom: Subtracted median distance maps upon KRAB recruitment where red indicates shorter pairwise distances, and blue larger ones compared to the no dox control. Data from five replicates are averaged. (**I**) Representative 3D chromatin structure (top), median distance maps (middle), and subtracted median distance maps (bottom) in irreversibly silenced (left) or reactivated cells (right). Data from four or six replicates are averaged. (**J**) Local radius of gyration (*R*_g_) is quantified at each genomic position using five consecutive segments (±10 kb), and its ratio compared to the no dox control is plotted as mean ± SEM.

We developed a system where we can control gene silencing and epigenetic memory (by recruitment and release of a chromatin regulator) and measure changes in chromatin state at the target locus and the region surrounding it. We used a synthetic reporter system (Fig. 1A) consisting of a fluorescent protein, Citrine, expressed under the control of a constitutive human EF1alpha promoter with 9x TetO recruitment sites (*27*). The reporter construct was stably integrated into the *AAVS1* safe harbor site on chromosome 19 in immortalized human embryonic kidney cells (HEK293T). Because HEK293T cells have three copies of chromosome 19 containing the *AAVS1* locus, multiclonal reporter lines may not correctly reflect fractional responses of the reporter. To exclude this confounding factor, we isolated clones that have single or double integrations (fig. S1, A to C, and see Materials and Methods). In these reporter lines, we introduced the KRAB repressor domain from ZNF10 fused to the reverse tetracycline repressor (rTetR) (*28*), facilitating precise control of recruitment and release through addition and removal of doxycycline (dox) to the culture media. After 5 days of KRAB recruitment, we observe complete silencing of the reporter (Fig. 1B, middle). Releasing KRAB from the target gene results in reactivation of gene expression in a subset of cells while the rest remain silenced, leading to a bimodal distribution of reporter expression levels that reaches steady state within 12 days after release (Fig. 1, B and C), similar to the all-or-none epigenetic memory formation previously described (*22*). Moreover, after dox removal and cell sorting, the silenced and reactivated states were stable over time, indicating that a fraction of cells becomes irreversibly silenced after KRAB recruitment (Fig. 1D).

To clarify if this all-or-none epigenetic memory results from subclonal heterogeneity, we sorted reactivated cells after memory establishment and repeated recruitment/release of the KRAB domain at the reporter (fig. S1D). We observed gene silencing and memory to a similar extent as the original population, confirming that the major source of heterogeneous all-or-none response is not genetically encoded, but rather epigenetically encoded. Moreover, the fluorescence intensity of the reporter in the reactivated cells was lower than in cells that were never silenced for the double integrant clone (fig. S1E). This can be explained by single-locus stochasticity where the reactivated population is a mixture of cells with one reactivated allele and cells with two reactivated alleles (fig. S1, F to I). This observation indicates that the silenced fraction of “cells” in the double integrant clone can be calculated as p^2^, where p is the silenced fraction of “loci”, not cells (fig. S1I). Hereafter, we report as epigenetic memory the silenced fraction of loci, the square root of the silenced fraction of cells in the double integrant clone, to more accurately compare this measurement to other analyses performed at the single-locus level, such as measurements of chromatin compaction.

To examine whether KRAB recruitment causes a change in 3D chromatin organization, especially the characteristic features of heterochromatin such as chromatin compaction or lamina association, we used a super-resolution microscopy-based chromatin tracing technique called Optical Reconstruction of Chromatin Architecture [ORCA; (*26*)]. ORCA relies on sequential hybridization and imaging steps in order to measure distances in units of nm between all points along the chromatin trace (Fig. 1E). To perform automated rounds of fluorescent probe hybridization, we added a custom-built liquid handling system to a commercial wide-field epifluorescence microscope (see Materials and Methods). To validate the performance of this system relative to the original ORCA system (*26*), we first visualized the TAD (topologically associating domain) and the distal enhancer-promoter interactions at the *MYC* gene locus (fig. S2). The median distance map generated by the ORCA system on a wide-field microscope showed a quantitatively similar profile to the one obtained on a previously reported ORCA system (fig. S2G; 0.9 correlation coefficient) and good agreement with Hi-C data (*29*) (fig. S2F; −0.88 correlation coefficient).

To measure the chromatin structure and compaction at our engineered *AAVS1* locus at the length scale associated with H3K9me3 spreading (tens of kilobases), we designed a DNA fluorescence in situ hybridization (DNA FISH) probe library spanning our integrated reporter and the 150-kb region around it. Each set of readout probes in the library targets a 5-kb segment: 13 contiguous segments (from −30 to 30 kb) around the reporter and three additional 5-kb segments upstream and downstream at 15-kb intervals (Fig. 1E). In addition, the entire region of interest is imaged in a second fluorescence channel in each round using fiducial probes as a reference for drift correction (*30*) (see Materials and Methods). Median distances measured with these probe sets are reproducible across replicates (fig. S2H).

We first examined the chromatin structure in silenced cells at the end of 5 days KRAB recruitment, as well as in irreversibly silenced and reactivated sorted cells. In all cases, the distance between DNA FISH spots and the edge of the nucleus did not change, indicating that the lamina association of the reporter locus does not increase significantly (Fig. 1F). In contrast, we find that the radius of gyration of the 3D chromatin polymer significantly decreases after 5 days of KRAB recruitment and in irreversibly silenced cells after KRAB release, but not in reactivated cells (Fig. 1G). The median distance maps between chromatin segments measured by ORCA also showed shorter distances in silenced cells compared to the no-dox cells (no recruitment, gene active) (Fig. 1, H and I). The KRAB EEW25AAA mutant, which is incapable of silencing gene expression or inducing epigenetic memory (*27*), served as a negative control and did not induce chromatin compaction upon recruitment with rTetR (fig. S3, A to C), suggesting that compaction is not caused by dox addition or rTetR binding to the TetO sites. In addition, we measured similar chromatin compaction in polyclonal HEK293T cells harboring Citrine and mCherry reporters (fig. S3, D and E) used in a previous study (*31*), suggesting that this observation is not restricted to a specific HEK293T clone. Most segment pairs that span across the reporter became closer upon KRAB-mediated silencing and in irreversibly silenced cells after KRAB release, unlike segment pairs that do not contain the reporter between them (fig. S4), suggesting that chromatin compaction is localized around the reporter integration region (fig. S4B). To estimate local chromatin compaction from the single-cell traces, we measured the radius of gyration around a ±10-kb region at each position and calculated the ratio upon KRAB recruitment (+dox) compared to no dox (Fig. 1J). This local ratio of radius of gyration decreased below one for regions within a range of tens of kilobases around the reporter, as expected in local compaction.

### Single-cell chromatin structure is heterogeneous even after complete silencing

**Fig. 2.**
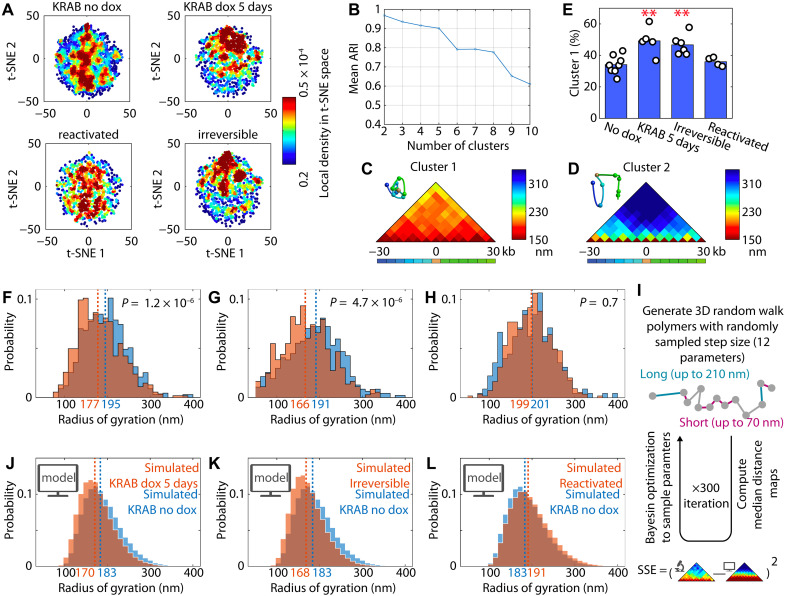
Single-cell chromatin structure is heterogeneous but shifted toward compacted conformations upon KRAB-mediated gene silencing. (**A**) Distribution of t-SNE values from each experimental condition. The scatter plot is color-coded by local density. All replicates for each condition are combined. (**B**) Mean ARI for a *K*-means clustering parameter sweep, with the number of clusters as the parameter. (**C** and **D**) Representative 3D chromatin structures and median distance maps of cluster 1 (C) and cluster 2 (D) at *K* = 2. (**E**) Proportion of loci in cluster 1 (with the compact conformation). Dots represent different biological replicates; each replicate quantified from 28 to 673 cells. **P < 0.01 by two-sided paired Student’s *t* test compared to no dox. Statistical comparison was made with only samples from the same replicate. (**F** to **H**) Representative radii of gyration distributions of single-cell traces for 5 days KRAB recruitment (F), irreversibly silenced (G), or reactivated (H) conditions in red, and no dox control in blue. *P* values from a Wilcoxon rank sum test are shown. (**I**) Schematic of the pipeline for Bayesian optimization to fit a 3D random-walk polymer model to experimental data (Materials and Methods). (**J** to **L**) Radii of gyration distributions from a 3D random-walk polymer simulation where the polymer step size was allowed to vary at each position to match the experimental median distance maps (fig. S6) for 5 days of KRAB recruitment (J), irreversibly silenced (K), or reactivated conditions (L) in red versus simulations fitted to the no dox polymer (blue).

Despite the clear difference in population-averaged distance maps, single chromatin traces are highly heterogeneous in both active and silenced cells (Fig. 1, H and I, top traces). Each chromatin structure may represent a distinct subpopulation in the set of possible conformations or alternatively arises from a continuous spectrum of chromatin structures. To perform an unbiased analysis of this heterogeneous, high-dimensional chromatin tracing dataset (78 dimensions, representing all combinations of pairwise segments within −30 to 30 kb), we first applied t-SNE dimensionality reduction to the aggregated data across all experimental conditions and then separated the individual conditions (see Materials and Methods). For each experimental condition, the projection of 3D chromatin configurations in this t-SNE space spreads broadly across all quadrants, showing largely overlapping distributions with and without KRAB recruitment (Fig. 2A). This suggests that no obviously distinct subpopulation of chromatin conformations is formed in the active state or upon silencing. We next performed *K*-means and Leiden clustering with different cluster numbers (*K*-mean) or Leiden resolution and measured average Adjusted Rand Index (ARI) to quantify the reproducibility of clustering (fig. S5, A and B, and see Materials and Methods). For *K*-means clustering, the average ARI was maximized at *K* = 2 and monotonically decreased as the number of clusters increased, suggesting that clustering with more than two clusters exhibits reduced reproducibility (Fig. 2B). The median distance maps for *K* = 2 reveal that the two clusters are either more compact or more open (Fig. 2, C and D). Furthermore, the proportion of traces belonging to the compact conformation cluster (cluster 1) significantly increases following 5 days of gene silencing mediated by KRAB recruitment, which is consistent with the t-SNE plots (Fig. 2E). Similarly, low reproducibility for more than two clusters was also observed with Leiden clustering (fig. S5, C and D, and see Materials and Methods). Collectively, these findings imply that the data are most accurately represented by a continuous spectrum with one major axis from compact to open, rather than well-separated, discrete states.

Consistent with the increased fraction of compacted conformations within the continuous spectrum, the single-cell distributions of the radius of gyration at the end of KRAB recruitment and in irreversibly silenced cells largely overlap with the no dox control, albeit with a smaller median (Fig. 2, F and G). In contrast, reactivated cells and no dox cells were not significantly different from each other (Fig. 2H). The continuous structure of the data and moderate compaction were further confirmed by rank plots (fig. S5E). To clarify how much of this heterogeneity could be the result of experimental noise, we developed a method to evaluate the degree of experimental noise. We assessed chromatin compaction before and after adding experimentally derived noise to chromatin traces with distinct configurations. We first measured the noise by rehybridizing one of the segments (#7) at the end of chromatin tracing (fig. S5F). We measured the difference between the *xyz* coordinates of the first and second hybridizations, fit these data with a *t*-location scale distribution, and sampled random values from this distribution to simulate experimental noise (fig. S5G). We classified chromatin traces into either “t-SNE low” or “t-SNE high” based on the t-SNE 2 value and then calculated their radius of gyration before and after adding noise to chromatin traces (fig. S5H). We found a clear segregation between t-SNE low and high populations even with simulated noise (fig. S5I), suggesting that the experimental noise could not explain the observed heterogeneity.

We next hypothesized that the observed heterogeneity could arise from the dynamic polymer nature of the chromatin fiber that samples many conformations. The dynamic nature of chromosome folding has been proposed before to explain the lack of distinct clusters of 3D chromosome conformations observed in single-cell data for CTCF-mediated chromatin looping (*32*) and long-range enhancer-promoter interactions (*33*). To analyze the extent of heterogeneity expected from a flexible fiber, we developed a simple 3D random walk polymer model (see Materials and Methods) and analyzed the predicted single-cell heterogeneity. In this model, we sampled step sizes (distance between nodes of polymers) between 70 and 210 nm and used Bayesian optimization to find the step sizes that match our experimentally measured median distance maps (Fig. 2I, fig. S6, and see Materials and Methods). The radii of gyration distributions of random walk polymers with fitted step sizes show a large overlap between polymers fitted to distance maps from silenced cells and those fitted to no dox conditions (Fig. 2, J to L), similar to the experimental data (Fig. 2, F to H). These results indicate that the heterogeneity of chromatin structure could arise from the flexible polymer nature of chromatin. In summary, even under stable active gene expression, chromatin at the tens of kilobase scale displays compact conformations without distinct subpopulations, and it becomes more biased, but not completely shifted, toward compact conformations upon silencing by KRAB recruitment.

### Chromatin compaction and histone methylation levels at the end of silencing quantitatively predict KRAB-mediated epigenetic memory

**Fig. 3.**
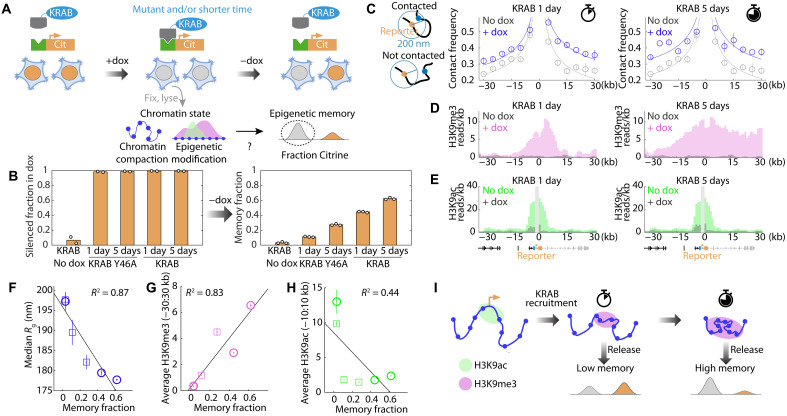
Repressive chromatin state predicts KRAB-mediated epigenetic memory. (**A**) Schematic representation of experimental workflow for quantifying epigenetic memory and chromatin compaction. (**B**) Silencing and epigenetic memory after varying recruitment durations for wild-type KRAB and Y46A mutant KRAB. Fraction of silenced loci was quantified in the B9 clone by RNA FISH at the end of recruitment (left) and flow cytometry for epigenetic memory 16 days after releasing KRAB (right). Memory fraction is defined as the square root of silenced fraction, assuming each allele stochastically establishes epigenetic memory (see fig. S1). (**C**) Contact frequency between the reporter-integrated region and other genomic regions measured by ORCA at the end of KRAB recruitment and in the no dox control. Circles show averages, with bars indicating SD. Lines show a power-law decay fit. (**D**) Genome traces showing normalized number of reads (averaged from two replicates) after CUT&RUN against H3K9me3 as a function of distance around the reporter integration site (at 0 kb) after KRAB recruitment (magenta) and in no dox control (gray). Traces are shown with 1-kb binning and moving average over 3-kb windows. (**E**) Genome traces showing normalized number of reads (averaged from two replicates) after CUT&RUN against H3K9ac as a function of distance after KRAB recruitment (gray) and in no dox control (green), analyzed and plotted as in (D). (**F** to **H**) The median radius of gyration for the −30- to 30-kb region around the reporter (F), normalized H3K9me3 CUT&RUN reads (G), and H3K9ac CUT&RUN reads (H) at the end of recruitment plotted against epigenetic memory. Circles (wild-type KRAB) and squares (KRAB Y46A mutant) show the average, with vertical and horizontal bars indicating the SD. Black lines show linear regression across conditions. (**I**) Schematic depicting chromatin state transitions during KRAB recruitment and epigenetic memory formation after release.

Having established the KRAB recruitment system and chromatin tracing methods, we set out to investigate the quantitative input-output relationship between the abundance of different epigenetic modifications, chromatin compaction, and KRAB-mediated epigenetic memory. We modulated the level of epigenetic memory in our system—the fraction of cells with irreversibly silenced Citrine reporters—by varying recruitment duration or by using a KRAB Y46A mutant with reduced memory. This KRAB mutant, which we identified as a reduced memory mutant in a previous functional screening assay (*27*), contains a point mutation (Y46A) in the A-box domain that binds to its main corepressor KAP1 (*34*). We silenced reporter expression with wild-type KRAB or KRAB Y46A for 1 and 5 days. At the end of recruitment, a portion of cells were fixed for chromatin tracing and chromatin modifications measurements, and the rest were cultured in dox-free media to measure epigenetic memory (Fig. 3A). Upon recruitment, regardless of the point mutation or the duration of recruitment, more than 90% of cells across all conditions are silenced as confirmed by RNA FISH (Fig. 3B, left). However, despite similar silencing profiles, we found different levels of epigenetic memory after dox removal, ranging from 10 to 60% across treatments (Fig. 3B, right), with 5 days of recruitment of wild-type KRAB having the highest fraction of irreversibly silenced cells and 1 day of recruitment of KRAB Y46A the lowest. Thus, the mutant KRAB or shorter recruitment times both decrease epigenetic memory measured 16 days after release, rather than affecting the level of gene silencing at the end of recruitment.

We proceeded to measure the chromatin states at the end of the recruitment phase to see if there was any difference in chromatin compaction and epigenetic modifications across the different conditions (despite them all being fully silent at that time) and if the chromatin states at the end of the silencing phase correlate with epigenetic memory after release. We observed increased contact frequency between the reporter and other chromatin regions within 1 day of KRAB recruitment and further increase particularly in the upstream region at day 5, suggesting progressive chromatin compaction (Fig. 3C). This was accompanied by gradual propagation of H3K9me3 (Fig. 3D), while H3K9ac showed confined enrichment around the reporter in the no-dox condition and was reduced to background levels within 1 day of recruitment (Fig. 3E). Similar recruitment duration-dependent compaction and H3K9me3 enrichment, as well as rapid deacetylation, were observed with KRAB Y46A mutant recruitment; however, chromatin compaction and H3K9me3 enrichment were less pronounced compared to wild-type KRAB recruitment (fig. S7, A to C). By measuring the radius of gyration across the −30- to 30-kb region around the reporter, we found that chromatin compaction at the end of recruitment correlated with the fraction of cells showing epigenetic memory after release (Fig. 3F and fig. S7D). Compaction, as measured by increased contact frequency or decreased radius of gyration, was not observed in nonsilencing controls (fig. S7, E and F). Similarly, H3K9me3 enrichment was also predictive of epigenetic memory (Fig. 3G). H3K9ac was consistently low across all conditions (except the no dox ones), reflecting efficient gene silencing (Fig. 3H). These results indicate that KRAB recruitment causes rapid loss of acetylation associated with gene silencing, while H3K9me3 accompanied by chromatin compaction accumulates more slowly and is correlated with epigenetic memory weeks later (Fig. 3I).

### After KRAB release, histone methylation is converted to DNA methylation, while chromatin compaction is maintained throughout the memory phase

**Fig. 4.**
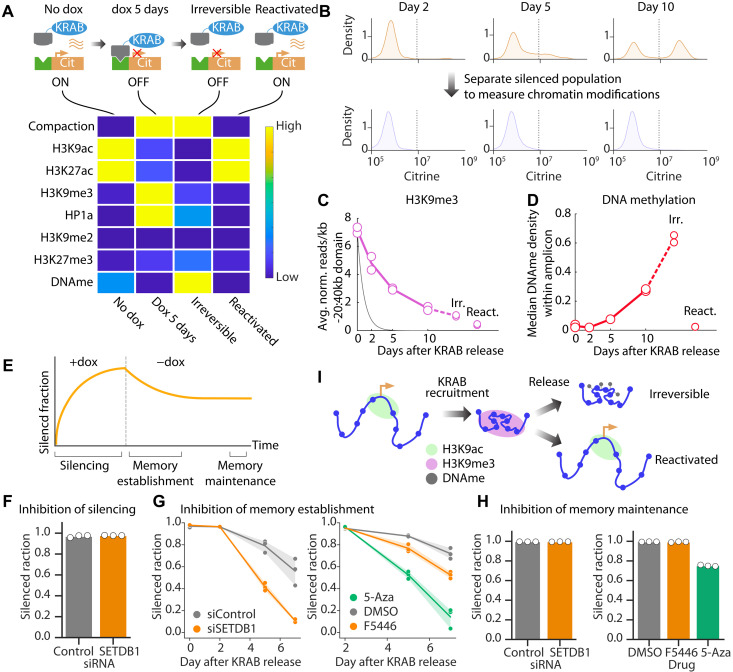
Targeted KRAB recruitment results in epigenetic memory formation mediated by DNA methylation. (**A**) Heatmap of normalized chromatin modifications abundances at the integrated reporter region. Histone and DNA modifications are measured using CUT&RUN and EM-seq, respectively (see Materials and Methods). Bar plots for each condition are presented in fig. S8. Compaction is defined as the absolute difference in the median radius of gyration between each condition and no dox control and is normalized such that the biggest difference (dox 5 days) is set to 1. (**B**) Histograms of Citrine intensity before and after magnetic separation of the silenced population after KRAB release. (**C**) Time course showing H3K9me3 CUT&RUN signal integrated over the −20:40-kb domain around the reporter. The gray curve shows theoretical dynamics due to passive dilution (see Materials and Methods). (**D**) Time course showing median DNA methylation density in reporter amplicon measured by EM-seq. (**E**) Inhibitor experiments were performed across three phases of gene regulation: silencing (concurrent with dox addition), epigenetic memory establishment (right after dox washout), and memory maintenance (on irreversibly silenced cells sorted before treatment). (**F**) Fraction of cells with Citrine silenced after 5 days of KRAB recruitment concurrent with siRNA-mediated gene KD of SETDB1 versus scramble siRNA control. Bars show the average of three replicates, each normalized to the no dox control. (**G**) Fraction of cells with Citrine remaining silenced during the KRAB release period (following 5 days of recruitment) upon addition of indicated siRNAs or inhibitors (5-Aza against DNA methylation and F5446 against H3K9 methyltransferase SUV39H1). Lines show the average of three replicates, each normalized to no dox. (**H**) Fraction of cells with Citrine silenced after 4 days of siRNA or inhibitor treatment of irreversibly silenced cells. Bars show the average of three replicates. (**I**) Schematic showing molecular transitions during KRAB-mediated epigenetic memory establishment.

To characterize chromatin changes during epigenetic memory maintenance, we measured histone modifications using CUT&RUN and DNA methylation with enzymatic methyl sequencing (EM-seq) at different time points after KRAB release. While KRAB recruitment resulted in H3K9me3 accumulation, most of the H3K9me3 enrichment was lost after KRAB release, not only in reactivated cells, but even in cells that remained irreversibly silenced for more than 30 days (Fig. 4A and fig. S8A). A similar trend was observed for HP1α enrichment, consistent with reported association with H3K9me3 (Fig. 4A and fig. S8B) (*35*). Both recently silenced (after 5 days of dox treatment) and irreversibly silenced cells are lacking active chromatin modifications H3K9ac and H3K27ac, consistent with complete gene silencing in these conditions (Fig. 4A and fig. S8, C and D). We did not detect other silencing histone modifications associated with gene silencing in other contexts, such as H3K9me2 or H3K27me3 (Fig. 4A and fig. S8, E and F) (*15*). Instead, we found DNA methylation enrichment at the reporter in the irreversibly silenced cells, but not at the end of KRAB-mediated silencing (5 days of recruitment) (Fig. 4A and fig. S8G). Chromatin compaction across tens of kilobases was a consistent signature throughout the molecular transition from histone to DNA methylation, being present in both recently silenced and irreversibly silenced cells (Fig. 4A).

To measure the dynamics of switching from H3K9me3 to DNA methylation within a silenced population after KRAB release, we separated silent cells using magnetic beads that bind to the cell surface marker coexpressed with Citrine (Fig. 4B) (*27*). Time-resolved H3K9me3 and DNA methylation profiling within the silenced population revealed that the H3K9me3 enrichment established at the end of 5 days of KRAB recruitment gradually decays after release, although there remains a small amount of residual H3K9me3 in the irreversibly silenced population (Fig. 4C). Meanwhile, DNA methylation slowly accumulates after KRAB release, reaching a maximum in irreversibly silent cells (Fig. 4D).

To investigate the molecular mechanisms required for epigenetic memory establishment and silencing in our system, we performed a series of knockdown (KD) and chemical inhibitor treatments during the silencing, memory establishment, and memory maintenance phases (Fig. 4E). Knocking down SETDB1, a major H3K9 methyltransferase and a member of the KAP1 repressor complex, did not impair repression during KRAB recruitment (silencing phase) (Fig. 4F). This lack of effect may be attributable to the involvement of other repression mechanisms mediated by the KAP1 repressor complex, such as histone deacetylation. In contrast, SETDB1 KD performed after KRAB release (memory establishment phase) substantially reduced epigenetic memory (Fig. 4G, left). Furthermore, inhibiting SUV39H1, another H3K9 methyltransferase, with the SUV39H1-specific inhibitor F5446 following KRAB release similarly resulted in reduced epigenetic memory (Fig. 4G, right). However, SETDB1 KD or F5446 treatment in the irreversibly silenced cells (memory maintenance phase) did not lead to reactivation (Fig. 4H). Collectively, these results suggest that H3K9me3 is required for epigenetic memory establishment, but not for memory maintenance. Treatments with a small interfering RNA (siRNA) mixture targeting the heterochromatin proteins HP1alpha, HP1beta, and HP1gamma or chemical inhibition of the RING1 from polycomb repressive complex 1 (PRC1) did not affect silencing, memory establishment, or maintenance (fig. S9). This lack of effect may be due to either insufficient KD or inhibition or because these molecules are not directly involved in this specific epigenetic gene silencing and memory formation process.

Consistent with the accumulation of DNA methylation after KRAB release, treatment with a DNA methyltransferase inhibitor 5-Aza-2′-deoxycytidine reduced epigenetic memory during the establishment phase and also reactivated irreversibly silenced cells (5-Aza; Fig. 4, G and H), suggesting that irreversible silencing is maintained by DNA methylation.

In summary, KRAB recruitment causes a 60-kb-scale H3K9me3-enriched domain, which slowly decays after release. H3K9me3 mediates gradual DNA methylation accumulation after KRAB release, which “locks in” the silenced state, while some cells stochastically reactivate the reporter before achieving stable silencing (Fig. 4I).

### Transcriptional silencing does not require nor produce chromatin compaction at the tens of kilobases scale

**Fig. 5.**
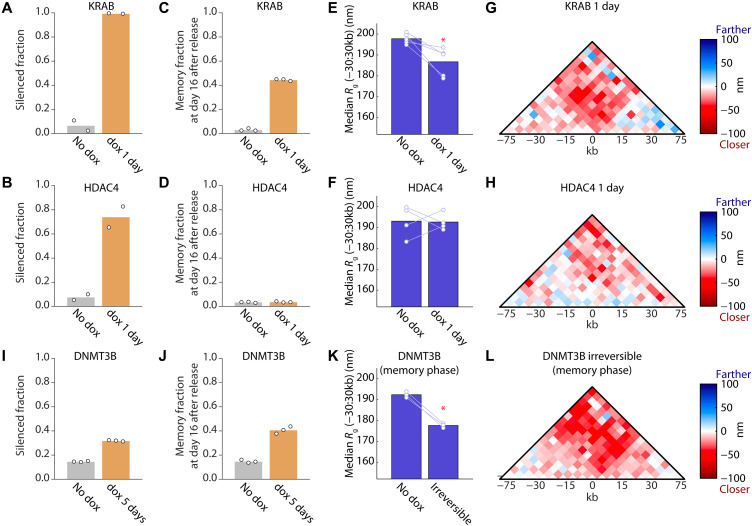
Loss of transcription does not lead to tens of kilobase-scale chromatin compaction. (**A** and **B**) Fraction of cells with the reporter silenced measured at the single-cell level by RNA FISH (B9 clone). No dox control is shown in gray, and KRAB (A) or HDAC4 (B) recruitment by addition of dox (1 μg/ml) for 1 day is shown in yellow. (**C** and **D**) Memory fraction at day 16 after the release of KRAB (C) or HDAC4 (D). Memory fraction is defined as the square root of silenced fraction of cells. (**E** and **F**) Median of the radius of gyration (from −30 to 30 kb) with or without KRAB (E) or HDAC4 (F) recruitment. *P < 0.05 by two-sided paired Student’s *t* test compared to no dox. (**G** and **H**) Subtracted median distance map between KRAB (G) or HDAC4 (H) recruitment for 1 day versus no dox. (**I**) Fraction of silenced loci measured by flow cytometry upon DNMT3B recruitment by addition of dox (1 μg/ml) for 5 days, defined as the square root of silenced fraction of cells. No dox control is shown in gray. (**J**) Memory fraction at day 16 after the release of DNMT3B, defined as the square root of silenced fraction of cells. (**K**) Median of the radius of gyration (from −30 to 30 kb) in the irreversibly silenced cells after DNMT3B recruitment and no dox control. *P < 0.05 by two-sided paired Student’s *t* test compared to no dox. (**L**) Subtracted median distance map between irreversibly silenced cells after DNMT3B recruitment versus no dox.

To gain a deeper understanding of the relationship between chromatin compaction and gene expression, we tested additional perturbations of chromatin and transcription and assessed their effect on 3D chromatin folding with ORCA. As an alternative silencing perturbation, we used the histone deacetylase HDAC4, which is known to remove acetyl groups from histones (*36*) and repress gene expression (*22*). Both KRAB and HDAC4 silenced reporter gene expression within 1 day of recruitment, as quantified by RNA FISH (Fig. 5, A and B). The H3K9ac profile upon HDAC4 recruitment shows more residual acetylation compared to KRAB recruitment, indicating that partial histone deacetylation is sufficient to inhibit transcription (fig. S10A). However, in contrast to KRAB recruitment, HDAC4 recruitment for 1 day did not exhibit epigenetic memory (Fig. 5, C and D) and did not lead to the clear chromatin compaction across tens of kilobases that is observed in KRAB recruitment, as measured by the radius of gyration and pairwise contacts (Fig. 5, E to H). These findings suggest that transcriptional silencing without memory does not require chromatin compaction at this length scale (10 to 20 kb) and that loss of transcription by itself does not cause the type of chromatin compaction over 10 to 20 kb we observed with KRAB recruitment.

Given that irreversibly silenced cells after KRAB release gain DNA methylation and retain chromatin compaction, we next tested if irreversible silencing via DNA methyltransferase DNMT3B recruitment leads to chromatin compaction. DNMT3B recruitment results in slow but permanent silencing, and the irreversibly silent cells showed an enrichment in DNA methylation levels at the promoter of the Citrine reporter gene (Fig. 5, I and J, and fig. S10B). Moreover, the irreversibly silenced population exhibited chromatin compaction (Fig. 5, K and L). H3K9ac was lost in the DNMT3B-mediated irreversible population, similarly to KRAB (fig. S10C). Note that H3K9me3 enrichment above background levels was not observed in the DNMT3B-mediated irreversible population (fig. S10, D and E). This suggests that DNA methylation deposition could independently cause chromatin compaction. Together, these results indicate that loss of transcription alone without memory does not induce chromatin compaction on the tens of kilobases scale, and instead, chromatin compaction on this scale appears in scenarios associated with memory.

### Chromatin spreading model suggests that chromatin compaction facilitates the histone-to-DNA methylation handoff during epigenetic memory establishment

**Fig. 6.**
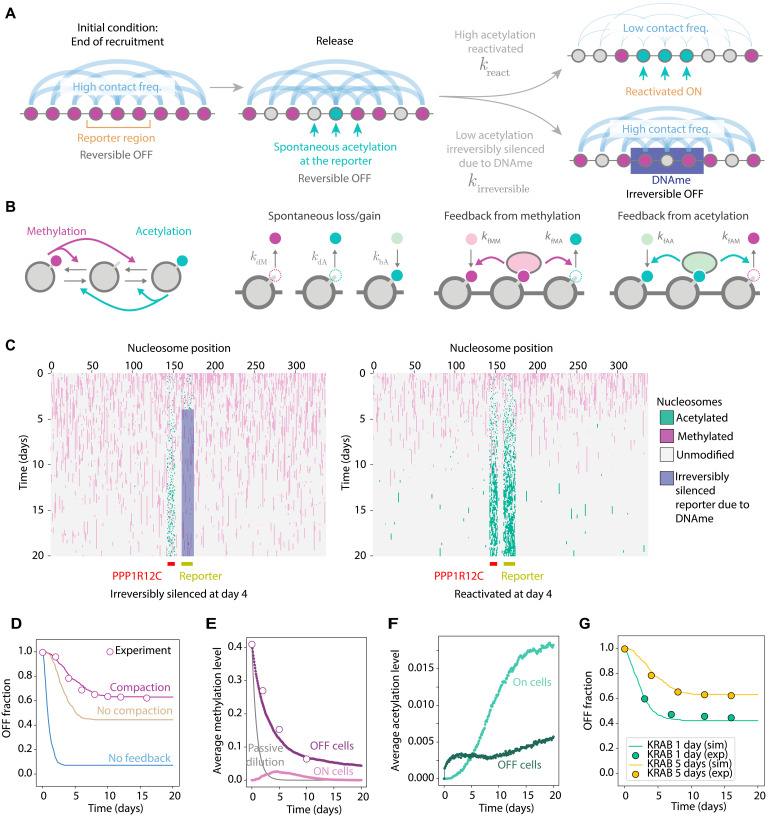
Stochastic simulation suggests that chromatin feedback and compaction facilitate DNA methylation-mediated epigenetic memory establishment. (**A**) Reporter locus is modeled as an array of nucleosomes (see Materials and Methods). During the KRAB release phase (middle) nucleosomes can lose methylation (gray circles) or gain acetylation (green circles) stochastically at the rates shown in (B), assuming single-locus stochasticity (fig. S12A). (**B**) Left: Schematic summarizing feedback among histone modifications. Middle and right: All allowed reaction rates during the release phase, with values listed in table S2, and distance dependence summarized in fig. S12B. (**C**) Representative simulation dynamics for a cell that committed to the irreversible OFF state (left) or reactivated ON state (right). Spontaneous acetylation is only allowed at the reporter (yellow bar) and the nearby promoter and first intron region of the endogenous PPP1R12C gene, matching CUT&RUN measurements (fig. S12C). Blue square indicates the irreversibly silenced reporter region that contains DNA methylation and does not allow spontaneous acetylation. (**D**) Time course showing the fraction of OFF cells from the simulation with compaction and feedback (purple) matching experimental data after 5 days of KRAB recruitment, without feedback reactions (blue), and with feedback reactions but no compaction (mustard). (**E** and **F**) Time course showing average histone methylation (E) or acetylation (F) levels over the entire nucleosome array simulation from the cells classified as ON (reactivated) or OFF (reversible and irreversible) as described in (A). Dots show average CUT&RUN H3K9me3 signals at each time point. The gray curve shows theoretical dynamics due to passive dilution (see Materials and Methods). (**G**) Time course showing the fraction of loci classified as an OFF state in the simulation. Simulations with the initial condition matching the end of 5-day and 1-day KRAB recruitment are shown in yellow and green, respectively. Dots show experimentally measured OFF fraction of loci by flow cytometry.

To dissect the relative contributions of feedback from chromatin modifications and 3D genome contacts to epigenetic memory in our system, we developed a stochastic simulation that incorporates our observation that, after KRAB release, H3K9me3 gradually decays and is replaced by DNA methylation in cells that retain memory (Fig. 6A). Although earlier simulations incorporated spreading of histone methylation due to reader-writer feedback and chromatin looping (*37*–*41*), they modeled scenarios where epigenetic memory is maintained through persistent histone methylation (as appropriate for biological systems lacking DNA methylation such as fission yeast) and therefore cannot account for the histone-to-DNA methylation handoff observed here. Moreover, our system uniquely provides time-resolved measurements of chromatin modifications, 3D contacts, and gene expression at the same locus, enabling an integrated modeling framework for memory establishment.

We model our system as an array of nucleosomes that start mostly in the H3K9me3 methylated state at the end of KRAB recruitment (Fig. 6A, left), matching H3K9me3 densities derived from CUT&RUN experiments (fig. S11A). During the release phase, we let the system evolve, allowing nucleosomes to lose histone methylation and gain acetylation (Fig. 6A, middle). In this model, we allow negative feedback between histone methylation and acetylation as well as positive feedback among modifications of the same type: The methylation of a given nucleosome promotes deacetylation and methylation of neighboring nucleosomes, whereas an acetylated nucleosome drives the opposite reactions (Fig. 6B). A locus that stochastically gains acetylation above a certain threshold can transition to a reactivated state that loses 3D compaction, whereas a locus with persistently low acetylation can transition to an irreversibly silenced, DNA-methylated state (Fig. 6A, right). The simulations reach two types of steady states: a stable, irreversibly silenced state (OFF) lacking acetylation and with some histone methylation retained across the nucleosome array (Fig. 6C, left) or a stable reactivated state (ON) that has lost histone methylation and gained histone acetylation (Fig. 6C, right).

To quantify the contributions of feedback and 3D contacts on epigenetic memory in the simulations, we compared three scenarios: (i) no read-write feedback, (ii) local neighbor-based feedback, and (iii) feedback modulated by 3D contact probabilities derived from single-cell chromatin tracing along the locus (fig. S11B). While read-write feedback was necessary for memory formation, incorporating increased spatial (3D) feedback due to chromatin compaction further increased the fraction of irreversibly silenced cells by ∼30% (Fig. 6D). This stochastic simulation also quantitatively captures the dynamics of chromatin modifications: In the OFF population, H3K9me3 decayed more slowly than expected from passive dilution and remained higher than in ON cells, matching experimental measurements (Fig. 6E), while acetylation showed the opposite trend (Fig. 6F). The measured H3K9me3 decay is consistent with weak read-write feedback that would not be sufficient to maintain memory in the absence of DNA methylation, even in a model that does not include the opposing histone acetylation (fig. S11) (*38*). Furthermore, simulations initialized with the distinct chromatin states generated by recruiting wild-type KRAB for either 5 or 1 day or by recruiting the Y46A KRAB mutant accurately captured the experimentally observed dynamics of irreversible silencing for each condition as well (Fig. 6G and fig. S12, D to F).

Together, these results suggest that chromatin compaction, through increased contact frequency, enhances the reader-writer feedback reaction and thereby facilitates long-term silencing memory establishment through the molecular handoff between H3K9me3 and DNA methylation.

### Chromatin compaction upon differentiation correlates with irreversible fate commitment in mESCs

**Fig. 7.**
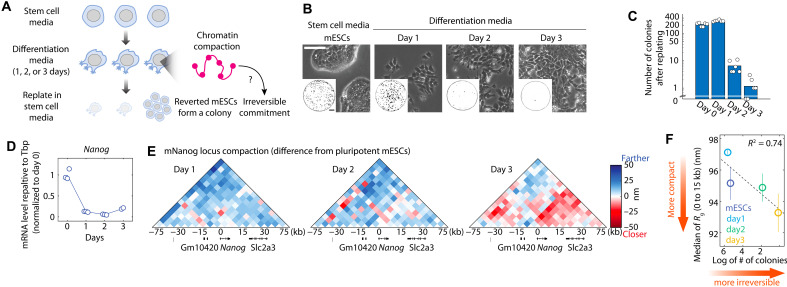
Chromatin compaction correlates with irreversible fate commitment in mESC differentiation. (**A**) Schematic representation of the experimental workflow for quantifying irreversible fate commitment and chromatin compaction during mESCs differentiation in N2B27 media. (**B**) Example images of mouse embryonic stem cells before differentiation and at different time points of differentiation. Scale bar (white), 100 μm. Insets show colonies after replating differentiated mESCs in stem cell media. Scale bar for insets (black), 250 mm. (**C**) Number of colonies after replating, calculated from insets in (B). (**D**) *Nanog* expression level was measured by reverse transcription qPCR. The mRNA levels were quantified relative to Tbp, then normalized to the average mRNA level at day 0. Line plots show the average across two or three biological replicates (dots). (**E**) Subtracted median distance maps upon differentiation compared to pluripotent mESCs. Data from two replicates are averaged. (**F**) Median radius of gyration of the 0- to 15-kb region is plotted against the log of the number of colonies in (B). Circles show the average of two replicates, and vertical bars show maximum and minimum values.

Next, we wanted to see if the predictive relationship between chromatin compaction at the end of silencing and irreversible commitment at a later time point can be observed in a natural biological process where genes are epigenetically silenced, for example, during cell differentiation. A recent study has demonstrated that gradual H3K9me3 enrichment at the *Nanog* gene locus is important for timing irreversible fate commitment during mESCs differentiation after 2i/LIF withdrawal (*42*). To modulate and assess the percentage of cells that are irreversibly committed, we initially grew cells in differentiation media for variable times and then replated 500 cells in stem cell media, allowing the ones that are not irreversibly committed to form ES colonies (Fig. 7A) (*42*). Cell morphology changed upon differentiation (Fig. 7B), and we confirmed previously reported changes in gene expression of cell marker genes (*42*, *43*) by quantitative polymerase chain reaction (qPCR) (fig. S13). In addition, we observed an increase over time in irreversible commitment to differentiated cells that cannot revert to mESCs, as evidenced by the decreasing number of cell colonies after replating (Fig. 7, B and C), consistent with previous findings (*42*). While the *Nanog* gene was silenced as early as day 1 (Fig. 7D), the chromatin structure around the *Nanog* locus exhibited gradual compaction over a longer time period (as observed with KRAB recruitment), with compaction increasing mostly between day 2 and day 3, consistent with reported gradual increase of H3K9me3 at this locus during differentiation (Fig. 7E) (*42*). Similarly to our KRAB-targeted reporter, this chromatin compaction could be measured as a decrease in the median radius of gyration at the *Nanog* locus, which correlated well with the decrease in the number of colonies (i.e., increase of cell fate memory) (Fig. 7F). To investigate if other genes could exhibit fast gene silencing and slow chromatin compaction dynamics, we reanalyzed a published dataset measuring gene expression, histone modifications, and chromatin 3D contacts by Hi-C during mESC differentiation to neuronal progenitor cells (NPCs) and subsequently to cortical neurons (CNs) (*44*). We looked for genes that were down-regulated in NPC and CN and displayed H3K9me3 enrichment in NPC and increased chromatin compaction scores in NPC and CN (see Materials and Methods). This approach identified 97 genes following such a pattern of fast down-regulation followed by more gradual compaction, including pluripotency transcription factors *Esrrb* (*45*), *Tbx3* (*46*), and *Gbx2* (*47*) (fig. S14 and data S1), although the exact timescale of the delay is hard to measure due to limited time points. Together, these results support a correlation between progressive chromatin compaction at multiple genes and irreversible fate commitment during mESC differentiation. Although this behavior parallels the dynamics observed in our synthetic silencing system, the extent to which endogenous loci undergo a similar H3K9me3-to-DNA methylation transition remains to be established.

## DISCUSSION

Combining a synthetic recruitment and release system and single-cell chromatin imaging, we investigated how epigenetic perturbations lead to changes in histone modifications and 3D chromatin organization, and how these changes connect to epigenetic memory at later time points. We show that KRAB recruitment leads to gene silencing, chromatin compaction (associated with H3K9me3 spreading) across tens of kilobases around the reporter gene, and stochastic long-term gene silencing memory after release. The level of compaction and H3K9me3 enrichment at the end of KRAB-mediated silencing quantitatively predict the fraction of cells with KRAB-mediated epigenetic memory after release. However, the molecular driver of gene silencing changes over time: in irreversibly silenced cells (2 weeks after KRAB release), the levels of H3K9me3 are substantially reduced at the locus and are instead replaced by DNA methylation. On the other hand, we do not observe this long-range chromatin compaction in cells that are silenced reversibly, suggesting that gene silencing alone is not sufficient to cause compaction at this length scale and that compaction is not necessary for silencing in this context.

We also showed a quantitative link between chromatin compaction and irreversible fate commitment during cell differentiation: Chromatin compaction at the *Nanog* locus during mESC differentiation correlates with the number of cells that are irreversibly committed to the differentiated state, and our analysis on published Hi-C data suggests that there are other gene loci whose compaction correlates with fate commitment. Chromatin changes associated with irreversible fate transitions have been reported in other systems: In a granulocytic differentiation model derived from murine bone marrow, irreversible fate commitment is associated with irreversible loss of chromatin accessibility measured by ATAC-seq (*48*). Moreover, genome-wide chromatin accessibility and gene expression analyses of hair follicle differentiation in mouse skin have shown that local chromatin accessibility changes before gene expression and foreshadows lineage choice (*49*). In fruit fly eye development, chromatin decompaction at the *ss* gene locus (imaged by DNA FISH) precedes R7 subtype differentiation (*50*). These studies suggest that chromatin structure at the end of an epigenetic perturbation or differentiation stimulus correlates with the potential of gene expression in the future. Genome-wide analyses of chromatin architecture (*51*, *52*) could be used to test predictions of irreversible fate commitment during differentiation to different cell types (before the end of the differentiation protocol) or during other processes associated with long-term epigenetic modifications, such as aging (*53*) or inflammation (*54*).

The effect sizes of chromatin compaction at the single-cell level were modest (in both the synthetic KRAB recruitment and the mESC differentiation systems), as evidenced by the small changes in the median gyration radii (Figs. 2 and 7) and substantial overlap in the distributions of gyration radii measured in the active and silent conditions (Fig. 2). This heterogeneity suggests that chromatin does not undergo an all-or-none transition from fully open to fully compact but instead is dynamic in both the active and silenced states. Thus, it is not likely that chromatin compaction at this length scale acts as a physical barrier to prevent transcription machinery from binding to the promoter. Similar single-cell results were recently reported for Polycomb repression in mESCs (*55*). Despite this single-cell heterogeneity we observe, differences in average chromatin compaction across conditions were substantial; in conditions that lead to high epigenetic memory, changes in the radius of gyration range from 0.8× to 0.9×, corresponding to reductions in chromatin volume by almost a half to a quarter (since 0.8^3^ = 0.512 and 0.9^3^ = 0.729) and therefore a major increase in nucleosome density. This magnitude is similar to direct measurements of nucleosome density using electron microscopy-based method, ChromEMT, which revealed that the density of heterochromatin is twice that of euchromatin (*56*).

In our system, H3K9me3 gradually propagates over 5 days during KRAB recruitment, and upon KRAB release, it decays and is converted into DNA methylation over a period of several days as the epigenetic memory is established and maintained. Similarly slow day-scale H3K9me3 propagation dynamics have been observed in mESCs (*13*) or during differentiation (*42*, *57*). We observed that DNA methylation increased mostly after the release of the KRAB domain. This is consistent with our previous observation that 18 days of continuous KRAB recruitment did not induce detectable DNA methylation at the reporter locus (*58*). These results suggest that the memory formation process in our system is mediated by an endogenous system that converts H3K9me3 to DNA methylation, not necessarily by cofactors associated with the KRAB domain.

Through chromatin spreading simulations, we find that read-write positive feedback on H3K9 methylation via 3D chromatin looping can recapitulate the slow decay of histone methylation observed experimentally and the stochastic transition to the irreversibly silenced state. Although previous simulation studies have mainly investigated strong feedback regimes where the system maintains an all-acetylation or all-methylation state (*21**,* *37**,* *38**,* *59*), we found that a weak feedback regime matches our experimental results better, fitting the measured H3K9me3-to-DNA methylation handoff. While theoretical studies using polymer simulations (*40**,* *55**,* *60**–**62*) have proposed that chromatin compaction could facilitate maintenance of histone methylation bistable domains, our results reveal another mode of memory establishment in which compaction simply slows down loss of histone methylation long enough to allow its conversion into DNA methylation.

While cross-talk between H3K9me3 and DNA methylation has been established (*13**,* *63**–**65*), we provide evidence that persistent gene silencing after KRAB release can be mediated solely by DNA methylation and that gain of DNA methylation during memory establishment can be mediated by H3K9me3. This is reminiscent of early embryonic development models, where the KRAB-containing repressor Zfp708 is only expressed in oocytes and zygotes, and its target sites show H3K9me3 peaks at the zygote stage while DNA methylation persists at later stages (*66*), suggesting that our observation may be relevant to the dynamics of transient KRAB-ZFP expression during embryonic development. However, persistent silencing after transient KRAB recruitment at endogenous loci depends on the local chromatin context (*67*), so it remains to be determined whether the memory mechanism at other loci is also enabled by an H3K9me3-to-DNA methylation handoff. Moreover, although we observe similar dynamics of gradual chromatin compaction at about 100 endogenous loci during irreversible cell fate commitment in mESC differentiation, direct evidence for a sequential transition from a large histone methylation domain to DNA methylation as a mechanism for epigenetic memory is currently limited to our synthetic reporter system. Testing its generality will require quantitative genome-wide time-course measurements of gene expression, H3K9me3, DNA methylation, and chromatin compaction during cell-state transitions. Testing the mechanism genome-wide also requires distinguishing epigenetic memory from ongoing active repression, which is straightforward in our synthetic system where we can untether KRAB but remains technically challenging at endogenous loci.

Overall, our findings show a quantitative correlation between chromatin compaction and epigenetic memory that is maintained over time even when the underlying molecular states change, and while our simulation provides a potential explanation, it does not necessarily imply a causal relationship. A key limitation is that feedback molecules can mediate both epigenetic modifications deposition and chromatin compaction, making it difficult to selectively perturb 3D chromatin organization without also perturbing epigenetic modifications. The lack of experimental validation for the causative role of chromatin structure in epigenetic gene regulation is a recognized gap in the field (*68*). Future studies could explore physically perturbing chromatin structure (*69*), rather than genetic perturbations to further investigate this relationship.

## MATERIALS AND METHODS

### Human cell culture and reporter cell line generation

HEK cells (HEK293T, Takara Bio, #632180) were cultured in Dulbecco’s modified Eagle’s medium (DMEM)–GlutaMAX media (Gibco, 10566024) supplemented with 1× Penicillin-Streptomycin-Glutamine (Gibco, 10378016) and 10% fetal bovine serum (FBS) (Omega Scientific, FB-15). The reporter cell line was generated in previous work (*70*) by cotransfecting cells with TALEN-L (Addgene, #35431), TALEN-R (Addgene, #35432), and pJT039 (AAVS1-9xTetO-pEF-IGKleader-Cas9site-hIgG1_FC-Myc-PDGFRb-T2A-Citrine-PolyA) reporter donor (Addgene, #161927) (*27*), followed by antibiotic selection with puromycin (0.25 μg/ml). To isolate single clones, cells were diluted at 100 cells/20 ml and then dispensed into a 96-well plate at 200 μl per well (∼1 cell per well). After 12 days of culture, wells with a single colony were selected and further expanded. We measured Citrine reporter expression levels using flow cytometry ZE5 (Bio-Rad) and extracted genomic DNA with QuickExtract DNA Extraction Solution (QE09050, Lucigen). PCR on the genomic DNA was performed to identify the presence of chromosomes with or without reporter integration (*71*). To verify the number of integrations, we extracted genomic DNA from 2 million cells using the DNeasy Blood & Tissue Kit (QIAGEN, 69504) and performed droplet digital PCR (ddPCR) through a service provided by MOgene (fig. S1B). Note that the ddPCR result is not always concordant with the results of the genomic PCR: For example, the B9 clone did not show a positive band for PCR on the wild-type chromosome, suggesting that all three copies of chromosome 19 have the reporter, whereas ddPCR suggested that the B9 clone has two integrations instead of three. As an alternative way to check the integration, we performed DNA FISH using ORCA probes (described in ORCA: probe design and synthesis section in detail) and calculated the percentage of loci with fiducial signal (detected across all chromosomes 19 in a cell with ∼3000 cy3 probes across the entire imaged region) that have a positive readout DNA FISH signal at the reporter (that detects the integrated reporter segment with lower efficiency since it only uses ∼150 cy5 probes). The percentage of reporter-positive chromosomes (fig. S1C) quantitatively matches the ddPCR results (fig. S1B); therefore, we concluded that A6 has a single integration; B9, B11, and D5 have two; and F5 has three integrations. One possible cause for the lack of signal in the genomic PCR for some of the double integrant clones could be the formation of indels that abolish primer binding near the TALEN cut site. The fluorescence intensity also varied; for instance, clone D5 was a double integrant but showed higher fluorescence intensity than B9 or B11. We flagged D5 as an aberrant clone and selected B9 for downstream analysis, as its fluorescence intensity was representative of the other clones. We also note our previous observation that the dynamics of reporter expression following KRAB-mediated silencing were generally reproducible across multiple CHO-K1 clones (*31*).

The wild-type KRAB domain from ZNF10, KRAB Y46A mutant, KRAB EEW25AAA mutant, and VP64 were individually cloned as fusions to rTetR(SE-G72P) (*28*) using the backbone from pJT126 lenti pEF-rTetR(SE-G72P)-3XFLAG-LibCloneSite-T2A-mCherry-BSD-WPRE (Addgene, #161926) digested with Esp 3I–HF. They were delivered by lentivirus (*27*). Wild-type KRAB, KRAB Y46A mutant, KRAB EEW25AAA mutant, and VP64-positive cells were selected by blasticidin S (10 μg/ml; Gibco, A1113903) and sorted by gating for mCherry-positive signal.

The pPB:PGK-H2B-mIFP-T2A-rTetR-DNMT3B1-polyA-zeo plasmid was transfected in the HEK293T reporter cell lines together with Super PiggyBac Transposase to obtain cells stably expressing rTetR-DNMT3B. Cells were selected in zeocin (60 μg/ml; ant-zn-05, InvivoGen), followed by cell sorting by gating for mIFP-positive signal. Cell sorting was performed twice to enrich mIFP-positive cell population.

The rTetR(SE-G72P)-HDAC4-T2A-mTurquoise plasmid was generated by PCR amplifying full-length human HDAC4 with appended Gibson homology arms from pLB37_PB_pGK-H2B-mIFP-T2A-rTetR-HDAC4-zeo (Addgene, #179440) using Q5 hot start polymerase (NEB, M0494S). A lentiviral backbone containing pEF1alpha-driven expression of 3xFLAG-tagged rTetR(SE-G72P) was then linearized by Esp 3I digestion, and the HDAC4 PCR fragment was annealed downstream into the open reading frame with Gibson assembly using NEB HiFi Master Mix (NEB, 2621L). After delivering the construct by lentivirus, HDAC4-positive cells were sorted by gating for mTurquoise positive.

To sort reactivated and irreversibly silenced cells after the KRAB recruitment and release, double reporter integrant cells (B9) were treated with dox (1 μg/ml; Tocris, 40902) for 5 days and then cultured without dox for 22 days. A population where both copies of the reporter reactivated (top ∼2% in fluorescence intensity) and a completely silenced population were sorted using the SH800 cell sorter (SONY).

For the validation of ORCA on a wide-field microscope, K562 cells were used. The cells were cultured in RPMI 1640 medium supplemented with penicillin/streptomycin/glutamine and 10% FBS.

### Inhibitors treatments and KD experiments

The catalog numbers and concentrations of inhibitors and siRNAs used for each experiment are listed in table S3. Inhibitors treatments and knockdown experiments were conducted across three distinct phases of epigenetic gene regulation dynamics: silencing, epigenetic memory establishment, and memory maintenance. For the silencing phase, inhibitors were added concurrently with dox treatment (1 μg/ml). For the epigenetic memory establishment phase, cells exposed to dox (1 μg/ml) for 5 days were washed twice in Dulbecco’s phosphate-buffered saline (DPBS), resuspended in inhibitor-containing culture media, and plated into 24-well plates. For the HP1 double-dose condition, cells were retreated with siHP1 on day 5 of release. For the epigenetic memory maintenance phase, irreversibly silenced cells sorted using cell sorter (SH800, SONY) were treated with each inhibitor for 4 days.

The Lipofectamine RNAiMAX Transfection Reagent (13778100, Invitrogen) was used for siRNA delivery following the manufacturer’s protocol. For HP1 knockdown, a mixture of siRNAs targeting HP1alpha, HP1beta, and HP1gamma was used. The siRNA designs for SETDB1 and HP1 were chosen based on (*72*) and (*73*), respectively. The concentration of 5-Aza-2′-deoxycytidine (Sigma-Aldrich, A3656-5MG) was determined based on (*24*).

### Flow cytometry

To silence reporter expression and measure epigenetic memory, cells were cultured in the presence of dox (1 μg/ml; Tocris, 40902). During the dox-treatment phase, culture medium was exchanged every day. For removing dox, cells were spun down and washed with 250 μl of DPBS (Genesee Scientific, 25-508) twice and then resuspended in culture media with no dox. Cells were passaged every 2 to 4 days. Before measuring fluorescence on flow cytometry, cells were resuspended in 1× Hanks’ balanced salt solution (Gibco, 14175095) supplemented with 1 mM EDTA (Boston BioProducts, BM-711) and bovine serum albumin (0.5 mg/ml; BIOTIUM, 22014). Flow cytometry was performed to measure Citrine, mCherry, or mTurquoise fluorescence intensity on ZE5 (Bio-Rad). Flow cytometry data were gated by forward and side scatter to select live single cells, as well as on mCherry or mTurquoise positive signal to select cells expressing the rTetR-fused chromatin regulators. To calculate the fraction of cells with epigenetic memory, cells with the Citrine fluorescence level lower than 15.5 in log scale were classified as silenced. Analyses were performed in MATLAB using Easyflow developed by Y. Antebi (https://github.com/AntebiLab/easyflow) or in Python using the Cytoflow package.

### ORCA: Probe design and synthesis

We designed ORCA probe sets as previously described (*26*, *30*). In brief, the barcoded oligos were designed on the sense strand to avoid the probes hybridizing onto mRNA. The algorithm selects probes without homology to human repetitive sequences and with GC contents within the range of 20 to 80%. Oligos that can hybridize to each other were also removed. The code is available at Zenodo (https://doi.org/10.5281/zenodo.7698979) and on the Boettiger Lab GitHub repository (https://github.com/BoettigerLab/ORCA-public). The probes were ordered from GenScript and amplified as previously described (*30*). Briefly, the primary probes were amplified using Phusion High-Fidelity PCR Master Mix with HF Buffer (NEB, M0531S) by monitoring the amplification on a qPCR machine (CFX Connect, Bio-Rad) to make sure amplification is in the linear range. Single-stranded RNA was synthesized from the PCR product using HiScribe T7 Quick High Yield RNA Synthesis Kit (NEB, E2050S). The single-stranded RNA was then converted into single-stranded DNA (ssDNA) using the Maxima H Minus Reverse Transcriptase (Thermo Fisher Scientific, EP0753) and used as primary probes. The concentration of primary probes was measured using the Qubit ssDNA Assay Kit (Thermo Fisher Scientific, Q10212), and it usually ranged between 80 and 180 ng/μl.

### ORCA: Cell culture on a coverslip

To minimize batch effects, we always plated dox-treated cells and no dox control cells on the same coverslip. To create isolated cultures on a coverslip, we made a polydimethylsiloxane (PDMS) multiwell device on top of the imaging slide as follows: Silicone elastomer base and curing agent (Sylgard 184; Dow Corning, 4019862) were mixed at 10:1 ratio and poured into a 10-cm dish. The dish was placed in a desiccator to degas the PDMS for 1 hour. The degassed PDMS was then left on a bench at room temperature to cure. We used the PDMS at least 1 week after curing to get electrochemically stabilized PDMS. A 3.17 cm (1.25 inch)–by–1.9 cm (0.75 inch) PDMS chunk was cut out from the cured PDMS, and then 13 of the 3-mm holes were created using a disposable biopsy punch (MedBlades). The PDMS multiwell was pressed onto a 40 mm coverslip (Bioptechs). To plate HEK293T cells, the bottom of each PDMS well was coated with 25 μl of 5% Matrigel (Corning, 356231) in DMEM-GlutaMAX + 1× penicillin/streptomycin/glutamine + 10% FBS in a 37°C tissue culture incubator for one hour after brief centrifugation to spin down the matrigel solution to the bottom of the wells in a 6-cm dish. Meanwhile, cells were trypsinized with 0.25% trypsin-EDTA (Thermo Fisher Scientific, 252-000-56) for 5 min, spun down, and resuspended at the density of 8000 cells/25 μl with or without dox. After removing the matrigel solution, 25 μl of the resuspended cells was loaded into each well and spun down at 700*g* for 1 min at room temperature. A piece of Kimwipes (Kimberly-Clark, 34155) wetted with 2 ml of water was placed in the 6-cm dish to humidify the dish. After confirming that cells are at the bottom of wells, the coverslip in a 6-cm dish was then incubated in the TC incubator for 24 hours. After fixing the cells the following day (detailed in the next section), the PDMS multiwell device was removed, washed with 70% ethanol and Milli-Q water, and air-dried to reuse in future experiments. For experiments involving more than 1 day of dox treatment, cells were cultured in the presence of dox in a culture dish before plating on the coverslip. To plate K562 cells, a 40-mm coverslip was precoated with 0.01% poly-l-lysine for 1 hour in the culture incubator.

### ORCA: Primary probe hybridization

We performed primary probe hybridization for ORCA experiments as previously described with some modifications (*26*, *30*). Cells were cultured in 25 μl of the culture media in the PDMS multiwell device and fixed by adding 25 μl of 8% formaldehyde (Electron Microscopy Sciences, 15714-S) in 1× PBS [final concentration (f.c.) 4%] for 10 min. The formaldehyde solution was then aspirated, and the PDMS multiwell device was carefully removed. Cells were further fixed with 2 to 4 ml of 4% formaldehyde for 10 min by completely submerging the coverslip in the formaldehyde solution, followed by three washes with 1× PBS (Invitrogen, AM9625). Hereafter, all treatments and washes were performed using 2 to 4 ml of each solution to thoroughly treat the coverslip unless otherwise noted. We also want to note that the solutions should not be poured directly onto the fixed cells to avoid disrupting the cells. For long-term storage of the fixed cells, the coverslip was washed with 70% ethanol once, submerged in 70% ethanol, sealed with parafilm, and stored at −20°C in a paper box. Before the following steps were performed, the coverslip was placed at room temperature for 10 min and washed with 1× PBS. Cells were permeabilized with 0.5% Triton X-100 (Sigma-Aldrich, T8787-50ML) in 1× PBS for 10 min, 1% SDS (Sigma-Aldrich, L6026-250G) in 1× PBS for 15 min, and 0.1 M HCl (Fisher Chemical, SA56-1) for 5 min at room temperature. Three washes with 1× PBS were performed after each Triton X-100, SDS, or HCl treatment. Cells were then treated with ribonuclease A (10 μg/ml; Thermo Fisher Scientific, EN0531) in 1× PBS for 1 hour in a 37°C tissue culture incubator. After three 1× PBS washes, cells were treated with hybridization #1 buffer [0.1% (v/v) Tween 20 (Sigma-Aldrich, P9416-50ML), 50% (v/v) formamide (Millipore, S4117), 2× SSC (Invitrogen, AM9763)] for 35 min at room temperature. Meanwhile, a 22 mm–by–22 mm coverslip (Santa Cruz Biotechnology, sc-363555) was coated with 150 μl of Sigmacote (Sigma-Aldrich, SL2-100ML) in a fume hood. After the hybridization #1 buffer was removed, 50 μl of hybridization #2 buffer [50% (v/v) formamide, 2× SSC, 0.1% (v/v) Tween 20, 10% (v/v) dextran sulfate (BP1585-100, Thermo Fisher Scientific)] containing primary probes (∼500 ng) was added onto the fixed cells, and carefully covered with the Sigmacote-coated coverslip. The coverslip was heated at 90°C for 3 min, placed in an empty tip box filled with water, and incubated at 47°C in an incubator for 12 to 16 hours. Next day, the coverslip was washed with 2× SSC (pre-warmed at 47°C) in the 47°C incubator for 10 min. The 22 mm–by–22 mm Sigmacote-coated coverslip was carefully removed, and the cells were washed with the prewarmed 2× SSC in the 47°C incubator for 10 min. The cells were further washed with room temperature 2× SSC twice, followed by a post-fix buffer treatment [8% (v/v) formaldehyde, 2% (v/v) glutaraldehyde (Thermo Fisher Scientific, 50-262-17) in 1× PBS] for 1 hour at room temperature. The cells were then washed with 2× SSC three times. For *Nanog* and *MYC* gene primary probe hybridization, the temperature for overnight incubation and subsequent washes with prewarmed 2× SSC was set to 42°C.

### ORCA: Sequential hybridization, imaging, and image analysis

We performed sequential hybridization as previously described with some modifications (*30*). After primary probe hybridization, the coverslip was mounted on a FCS2 chamber (Bioptechs, 03060319-2-NH). The FCS2 chamber was connected to a home-built fluidics system controlled by home-built software and placed on the Leica DMi8 wide-field microscope imaging stage. First, 25% ethylene carbonate (EC; Sigma-Aldrich, E26258-3KG) in 2× SSC buffer containing 10 nM fiducial cy3 probes was perfused into the chamber and incubated for 15 min at room temperature. The sample was then washed with a wash buffer [30% (v/v) formamide in 2× SSC] and 2× SSC, and then an imaging buffer [glucose oxidase (0.25 mg/ml; Sigma-Aldrich, G2133-50KU), catalase (40 μg/ml; MP Biomedicals, 02100402-CF) and 9% (v/v) glucose (Sigma-Aldrich, G8769-100ML) in 2× SSC] was perfused in the chamber. Multiposition acquisition was manually set using the LAS X Leica microscope control software. The cy5 readout probes were prepared in a 96-well plate so that each well contains 100 nM adapter oligo, 110 nM cy5 readout oligo, and 300 nM displacement oligo to displace the previous round of hybridization, each in 750 μl of 25% EC buffer. The 96-well plate was covered with foil and placed on the fluidics together with ∼50 ml of wash buffer, 2× SSC buffer, and 20 ml of imaging buffer. The imaging interval time was set using the home-built software. The automated fluidics was started first, and then imaging was started immediately after the first round of hybridization finished. Imaging was performed with a 63×/numerical aperture (NA) 1.4 oil-immersion objective that generates 2048 pixel–by–2048 pixel images at the scale of 103 nm/pixel and using 200-nm z steps to scan 6 μm vertically. For the fluorophore excitation, we used Lumencor light-emitting diode illumination with 50% attenuation. The exposure times were 100 and 10 ms for cy5 and cy3, respectively.

To perform image analysis, we used ChrTracer3 developed by Boettiger Lab (*30*). The Leica-generated tif images were converted to dax format using a home-built software so that the files could be processed with ChrTracer3. The ChrTracer3 algorithm fits each fiducial and readout spot with a 3D Gaussian to get 3D coordinates. The fiducial coordinates were used to correct 3D drift. Chromatin traces were obtained from the readout coordinates after drift correction. We also filtered readout spots by the median distance to other readout signals (cutoff of 600 nm for *AAVS1* and *Nanog*, 1000 nm for *MYC*) to eliminate nonspecific background noise. To calculate the radius of gyration or perform t-SNE dimensionality reduction and *K*-means or Leiden clustering, traces were filtered to ensure segment detection efficiency above 50%, followed by linear interpolation to obtain complete 3D structures. To calculate the correlation between chromatin compaction and epigenetic memory or irreversible fate commitment or perform statistical analysis such as Student’s *t* test or Wilcoxon rank sum test, data from the same coverslip were used to minimize the impact of batch-to-batch variability when analyzing chromatin traces. To calculate the distance between the edge of the nucleus and the DNA FISH spots, cell masks were created using StarDist (*74*). The minimum distance between the pixels on the edge of the mask and the *XY* coordinates of the detected DNA FISH spots was then calculated.

To perform parameter sweeping and calculate the reproducibility of clusters, the median distances of pairwise segments were first log-transformed and *z*-scored. Principal components analysis was then performed, and PC scores that explained 90% of the variance were used for subsequent clustering analysis. The clustering algorithm (*K*-means or Leiden) was run 15 times on 80% of the randomly chosen dataset. ARI was calculated for each pair of runs and then averaged across all pairs (mean ARI). ARI calculates the fraction of data pairs (pairs of chromatin traces in this case) in agreement across different runs and corrects the score for random chance (*75*), serving as an indicator of clustering reproducibility.

To compare chromatin traces at the *MYC* locus with Hi-C data, we used Hi-C data previously reported (*29*). Normalized counts from the Hi-C data were exported as a matrix using JuiceBox (*76*). The exported matrix was further analyzed using custom scripts in MATLAB.

### RNA FISH for reporter mRNA quantification

RNA FISH probes for the reporter mRNA were designed the same way as the ORCA primary probes, except that they were designed on the basis of the antisense strand so that they hybridize to the mRNA. Cells (3 × 10^4^) in 20-μl culture media were plated in the matrigel-coated PDMS multiwell device for 10 min and then 20 μl of 8% formaldehyde in 1× PBS (4% at a final concentration) was added to fix the cells without removing culture media. After removing the PDMS multiwell device, cells were permeabilized with 0.5% Triton X-100 in 1× PBS for 10 min at room temperature. After three 1× PBS washes, cells were treated with hybridization #1 buffer for 35 min at room temperature. After the hybridization #1 buffer was removed, 50 μl of hybridization #2 buffer containing 200 ng of RNA FISH probes was added to the fixed cells and carefully covered with a 22 mm–by–22 mm Sigmacote-coated coverslip (as described in the ORCA protocol). The coverslip in a 6-cm dish was placed in an empty tip box filled with water and incubated at 37°C in an incubator for 12 to 16 hours. Next day, the coverslip was washed with 2× SSC (prewarmed at 37°C) in the 37°C incubator for 10 min. The 22 mm–×–22 mm coverslip was carefully removed, and the cells were washed with the prewarmed 2× SSC in the 37°C incubator for 10 min. The cells were further washed with room temperature 2× SSC twice. The RNA FISH signal was visualized using an adapter oligo that binds to the RNA FISH primary probes and has three tandem binding sites for the cy5 readout oligo. RNA FISH images were taken with a 63×/NA 1.4 oil-immersion objective using 200-nm steps z-scanning. 4′,6-diamidino-2-phenylindole (DAPI) staining images were taken after the RNA FISH imaging. Background was subtracted from the RNA FISH images using rolling ball background subtraction at the radius of 5 pixels. Then, the maximum intensity projection across the z-stack of images for a given XY position was used to generate single *XY* images. The number of RNA FISH spots was quantified using the spot detection algorithm in ImageJ with the prominence parameter set to 400. Cell masks were created using the StarDist plugin (*74*) with the following parameters: 'modelChoice':'Versatile (fluorescent nuclei)', 'normalizeInput':'true', 'percentileBottom':'20.0', 'percentileTop':'95.0', 'probThresh':'0.6', 'nmsThresh':'0.01', 'outputType':'Label Image', 'nTiles':'1', 'excludeBoundary':'2', 'roiPosition':'Automatic'. We filtered out cells with abnormal size and then classified cells with less than 25 RNA FISH spots as silenced based on the FISH distribution for cells with KRAB recruited for 5 days (+dox) that are fully silenced by flow cytometry.

### CUT&RUN for detection of histone modifications

CUT&RUN to profile histone modifications was performed as previously described using the CUTANA ChIC/CUT&RUN Kit (EpiCypher, 14-1048) and H3K9me2 antibody (Abcam, ab1220), H3K27me3 antibody (Cell Signaling Technology, 9733S), H3K9ac antibody (Abcam, ab4441), H3K27ac antibody (Active Motif, 39133), HP1α (Cell Signaling Technology, 2616S), and H3K9me3 antibody (Diagenode, C15410193) (*31*). An input of 5 × 10^5^ HEK293T cells was processed according to the manufacturer’s protocol. The double integrant clone (B9) was used for this experiment. Digitonin was used at a final concentration of 0.025% for nuclear permeabilization. Dual-indexed sequencing libraries were made using the NEBNext Ultra II DNA Library Prep Kit for Illumina (New England Biolabs, E7645S) or CUTANA CUT&RUN Library Prep Kit with Primer Set 1 (EpiCypher, 14-1001), and library concentrations were quantified with the Qubit 1 X dsDNA HS Assay Kit (Invitrogen, Q33231). Library fragment sizes were checked with Agilent 2100 Bioanalyzer through the Protein and Nucleic Acid Facility at Stanford University School of Medicine. For sequencing, a NextSeq System (Illumina) was used. A custom human genome (version hg38) with the reporter integration was constructed using bowtie2-build. Paired-end alignment was performed with a bowtie2 command, and duplicate reads were removed using Picard. Bedgraph files were generated from the reads, normalized by dividing by total counts in each sample, and reported as counts per million. Further analyses and visualization were performed using MATLAB. For H3K9ac and H3K27ac quantification, reads aligned to human EF1alpha promoter were excluded because they also include signal from the endogenous EF1alpha promoter or the EF1alpha promoter that drives expression of rTetR-fused chromatin regulators in addition to the target EF1alpha in the reporter. In Fig. 4A, signals are normalized such that negative controls are 0 and positive controls are 1. H3K9ac and H3K27ac are normalized to glyceraldehyde-3-phosphate dehydrogenase (GAPDH) (positive) and ZNF14 (negative), H3K9me3 and HP1α are normalized to CXCL12 (positive) and GAPDH (negative), H3K9me2 and H3K27me3 are normalized to DSCR4 (positive) and GAPDH (negative), and DNA methylation was normalized to 12 days of DNMT3B recruitment (positive) and DNMT3B no dox (negative).

### EM-seq for detection of DNA methylation

EM-seq was performed to profile DNA methylation. Briefly, 1 × 10^6^ HEK293T cells were collected, and their genomic DNA (gDNA) was extracted using the NEB Monarch Kit (NEB, T3010S) according to the manufacturer’s protocol. gDNA was subjected to restriction enzyme digestion with Xba I and Acc I for 1 hour at 37°C. Following SPRIselect purification (B23318, Beckman Coulter), digested DNA was converted using the New England Biolabs’ Enzymatic Methyl-seq Kit (E7120S, NEB) to detect selectively oxidized unmethylated cytosines. After conversion, three PCRs were performed to selectively amplify a segment of 771 base pairs (bp) of reporter DNA overlapping the pEF promoter. First, NEB Q5U polymerase (NEB, M0515) was used according to the manufacturer’s specifications to amplify reporter DNA from the pool of gDNA while maintaining the identity of converted uracils. Subsequently, amplified DNA was amplified again using NEB Q5 Ultra II to add Illumina read 1 and read 2 overhangs (NEB, M0544). Last, unique sequencing indices were added to each sample in a third PCR reaction also using NEB Q5 Ultra II. For sequencing, a MiSeq System (Illumina) was used with a 600v3 kit, reading 374 cycles in read 1 and 235 cycles in read 2. BWAmeth was used for methylation-specific alignment to the reporter, and Samtools was used to convert raw alignment files to indexed .bam. Bulk CpG methylation levels were calculated per site using MethylDackel (https://github.com/dpryan79/MethylDackel). Custom analyses were used (dSMF-footprints_optional_clustering.py) to produce single-molecule methylation heatmaps of aligned reporter molecules. Further analyses and visualization were performed using MATLAB.

### mESC culture and differentiation

Mouse embryonic stem cells CASTx129 were cultured in 2i + LIF serum-free media (stem cell media). The medium was made by mixing 0.5× DMEM/F12 (Gibco, 11320-033), 0.5× neurobasal medium (Thermo Fisher Scientific, 21103049), 1× GlutaMAX supplement (Thermo Fisher Scientific, 35050061), 0.05% bovine albumin fraction V (7.5% solution; Thermo Fisher Scientific, 15260037), 100 μM 2-mercaptoethanol (50 mM; Thermo Fisher Scientific, 31350010), 0.5X N-2 supplement (100×; Thermo Fisher Scientific, 17502-048), 1X penicillin/streptomycin 100× (Thermo Fisher Scientific, 15-140-122), and 0.5× B-27 supplement (50×) serum free (Thermo Fisher Scientific, 17504044) supplemented with 1× LIF 10000X (0.1 mg/ml; Miltenyi Biotec, 130-095-777), 1× 5 mM PD0325901 5000X (Axon Medchem, 1408), and 1× 15 mM CHIR99021 5000X (Axon Medchem, 1386). A culture dish was precoated with 0.0015% poly-l-ornithine (Sigma-Aldrich, P4957-50ML) for 1 hour in a 37°C tissue culture incubator followed by Laminin (10 μg/ml; Sigma-Aldrich, L2020-1MG) coating in the same way. For passaging, cells were trypsinized for 5 min with 0.25% Trypsin-EDTA (Thermo Fisher Scientific, 252-000-56), spun down after deactivation with equal amount of the stem cell media, and resuspended in fresh media because the leftover of trypsin inhibits cell adhesion. Cells were plated at a density of 3 × 10^5^ cells per well in a six-well plate. The stem cell medium was exchanged every day and cells were passaged onto a new plate every other day.

To differentiate mESCs, stem cell media without LIF, PD0325901, and CHIR99021 were used as the differentiation media (N2B27 media). Cells were plated at a density of 1 × 10^5^ cells per well in six-well plates in 2-ml differentiation media. After 1, 2, or 3 days of differentiation, 1 × 10^5^ trypsinized cells were spun down and frozen at −80°C for qPCR. For subsequent ORCA measurements, 2 × 10^5^ trypsinized cells were spun down and resuspended in 100 μl of 4% formaldehyde in 1× PBS for 10 min. These fixed cells were then spun down and resuspended in 30 μl of 70% ethanol and stored at −20°C until they were used for ORCA measurements. For use in the colony-based irreversibility assay, 2 × 10^3^ trypsinized cells were spun down and resuspended in 100-μl culture media, spun down again, and resuspended in 200-μl stem cell media. Of these, 500 cells in 50 μl were added to 500-μl stem cell media in 24-well plates coated with 0.0015% poly-l-ornithine and Laminin (10 μg/ml) and grown further for 5 days.

### mESC differentiation irreversibility assay based on colony formation

Mouse embryonic stem cells replated in the stem cell culture media after differentiation (see the “mESCs culture and differentiation” section) were fixed by adding 500 μl of 8% formaldehyde in 1× PBS, followed by 1× PBS wash twice. Cells were incubated with DAPI (1 μg/ml; AC202710500, Thermo Fisher Scientific) for 10 min, and each well was scanned with a 4× objective on the Leica DMi8. To estimate the number of colonies that originated from each individual dedifferentiated cell, images were processed as follows using ImageJ: A median filter with a radius of 4 was applied to all images, and then rolling ball background subtraction at a scale of 20 was performed. A Gaussian filter with sigma of 4 was applied, and then the images were binarized by Li thresholding. Pixel clusters were filtered to keep only the ones with a size of 40 to 10,000 pixels. Watershed was applied to further separate colonies from background noise (especially those close to the edge of the well). A second pixel cluster filtering was performed to select clusters with sizes between 40 and 2500 pixels and with circularity of 0.7 to 1.0, which were defined as colonies.

### Reverse transcription qPCR of fate marker genes

For each differentiation condition, 1 × 10^5^ cells were spun down and stored at −80°C as a cell pellet after removing culture media. After thawing, the total RNA was extracted using the RNeasy Mini Kit (QIAGEN, 74106). After quantifying ssRNA concentration using NanoDrop, the cDNA synthesis was performed using iScript Reverse Transcription Supermix (Bio-Rad, 1708840). For qPCR, the SsoAdvanced Universal SYBR Green Supermix (Bio-Rad, 1725270) was used. We used previously reported primer sets (*42*). CFX Connect (Bio-Rad) was used for the qPCR readout. The Tbp gene was used as an internal normalization control, and mRNA levels for each gene relative to Tbp were defined as 2^(Cq_Tbp - Cq_gene)^ and then normalized to the day 0 mRNA level so that the average mRNA at day 0 is equal to 1.

### ORCA: Mouse ES cells

Before plating the fixed ES cells suspensions onto a 40-mm coverslip, 5 μl of 0.01% poly-l-lysine was added in the PDMS multiwell device (each with a 3-mm inner diameter) pressed onto a 40-mm coverslip and completely air-dried for 1 hour in a tissue culture hood. 7.5 μl of ES cells suspensions fixed in 70% ethanol (as described in the “mESC culture and differentiation” section) was added into the PDMS wells and incubated for 10 min at room temperature. The 70% ethanol was removed and the PDMS multiwell device was carefully peeled off. After air-drying for 1 min, cells were fixed with 4% formaldehyde in 1× PBS for 10 min. The rest of the primary probe hybridization protocol was the same as described in the “ORCA protocol: Primary probe hybridization” section. We performed sequential hybridization as described above. To make this chromatin tracing comparable to the experiment performed at the synthetic reporter gene, we targeted a 60-kb region around the *Nanog* gene promoter at a resolution of 5 kb and three additional 5-kb-long segments upstream and downstream at 15-kb intervals.

### 3D random-walk polymer model fitting

We used a Bayesian optimization algorithm to fit a locally compacted 3D random-walk polymer model to the experimental data (Fig. 2I). Analysis was performed using Python on Google Colaboratory Pro. The CuPy package (*77*) was used to parallelize the computation on multiple GPUs, and the Optuna package (*78*) was used for Bayesian optimization. In each trial, 50,000 3D random-walk polymers were generated with randomly sampled step sizes. The parameter (step-size) search range was set to 70 to 210 nm. The TPE sampler was used to optimally sample the random-walk step size, with the prior_weight parameter set to 1000. The objective function to be minimized was defined as the sum of squared errors between the median distance maps measured experimentally and the median distance maps computed from the simulated 3D random-walk polymers. Trials were repeated 300 times, and the parameter set (of step sizes at each position) with the least error was chosen as the best fit. Ten independent optimizations were performed and the median of the best fit parameter sets was used for further calculations (e.g., radius of gyration).

### Stochastic simulation of histone modifications

Histone methylation and acetylation dynamics were simulated on a 1D array of 338 nucleosomes, corresponding to the integrated reporter and a ±30-kb region around the reporter. The initial condition was set by randomly setting each nucleosome in the nucleosome array to be methylated according to the normalized H3K9me3 density derived from CUT&RUN. Each nucleosome could be unmodified, methylated, or acetylated. The passive dilution rate of histone methylation was determined on the basis of the doubling time of HEK293T cells, measured from published live-imaging data of long-term HEK293T cell culture (*24*), with an average doubling time of 15 hours. In addition to the spontaneous loss of H3K9me3, we defined a feedback reaction where a reader-writer module binds to a methylated nucleosome and converts nearby unmodified nucleosomes to the methylated state. The unmodified nucleosome was chosen based on the looping probability between methylated and unmodified nucleosomes. Looping probability was defined as a linearly interpolated contact frequency matrix from ORCA measurements with a 200-nm threshold. We also defined a deacetylation feedback mechanism, where a reader-writer module on methylated nucleosomes converts nearby acetylated nucleosomes to the unmodified state, with the acetylated nucleosome chosen based on looping probability. Acetylation followed the same feedback mechanism but with different rates. The spontaneous gain of acetylation was set to occur in regions corresponding to pEF and Citrine within the reporter (15 nucleosomes) and the PPP1R12C promoter and the first intron (10 nucleosomes), matching our CUT&RUN data. During the release phase, cells start as reversibly OFF and can stochastically transition to an irreversible OFF state if the acetylation density across the reporter is lower than a set threshold. Once in this state, they are considered DNA-methylated at the reporter and cannot gain acetylation anymore in this region. Conversely, if the acetylation at the reporter is higher than the threshold, then cells can transition to a reactivated state where their contact probability decreases (to levels of those measured before recruitment). See table S2 for parameter values for all the simulation parameters. For each condition, 500 independent simulation runs were performed. The stochastic simulation was implemented using the Gillespie algorithm (*79*), using Python, parallelized on GPU with the CuPy package, and performed on Google Colaboratory.

### Hi-C data analysis

To analyze a published dataset of RNA sequencing, H3K9me3 chromatin immunoprecipitation sequencing (ChIP-seq), and Hi-C during mESC differentiation to NPCs and subsequently CNs (*44*), we selected genes whose expression levels in Fragments Per Kilobase of transcript per Million (FPKM) decreased by at least 50% in NPC and CN compared to the ES cells. H3K9me3-enriched loci in NPC were defined as those whose integrated H3K9me3 ChIP-seq signal within a 4-kb window around their transcription start site (TSS) is in the top 50% quantile. Chromatin compaction score was defined as the sum of Hi-C read counts in a 30-kb window around the TSS, omitting pairs less than 5 kb apart. This score was normalized to the median value of all genes for each condition. Loci were further filtered if there was a 20% increase in the chromatin compaction score in NPC or CN compared to the ESCs, with a bigger increase in CN than in NPC. The analysis was conducted using Python. MATLAB was used for visualization.

## Supplementary Materials

### The PDF file includes:

Figs. S1 to S14 Legends for tables S1 to S3 Legend for data S1 References
