# Supplementary material for: Single-cell chromatin state transitions during epigenetic memory formation
Source: Sci Adv. 2026 Jul 23;12(30):eaeb0060. doi: 10.1126/sciadv.aeb0060 (PMC13394403; doi:10.1126/sciadv.aeb0060)
Supplement: Supplementary file 1 — Figs. S1 to S14 Legends for tables S1 to S3 Legend for data S1 References [file sciadv.aeb0060_sm.pdf]

Science Advances
AAAS

Supplementary Materials for

Single-cell chromatin state transitions during epigenetic memory formation

Taihei Fujimori *et al.*

Corresponding author: Lacramioara Bintu, lbintu@stanford.edu

Sci. Adv. **12**, eaeb0060 (2026)
DOI: 10.1126/sciadv.aeb0060

The PDF file includes:

Figs. S1 to S14
Legends for tables S1 to S3
Legend for data S1
References

Other Supplementary Material for this manuscript includes the following:

Tables S1 to S3
Data S1

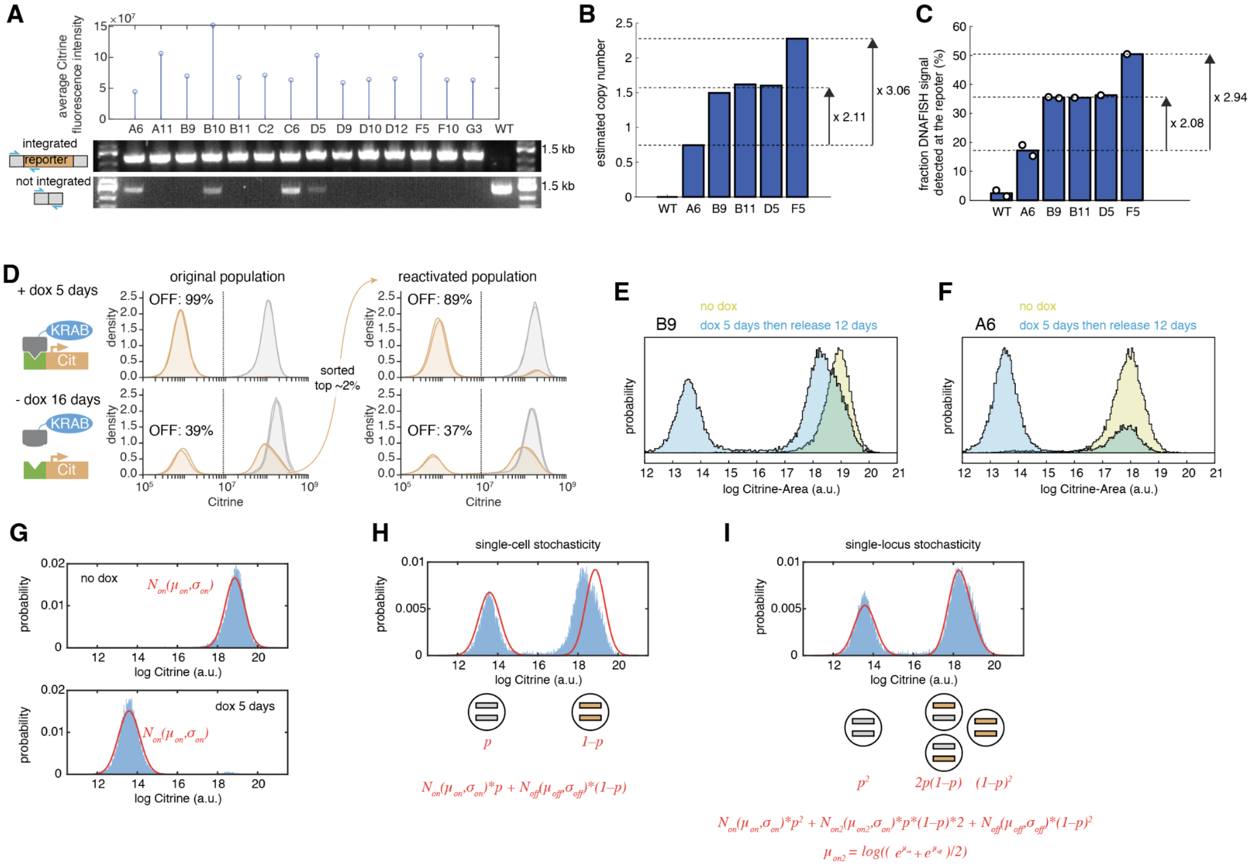

Fig. S1. Epigenetic memory for cell clones with different numbers of reporter integrations.

(**A**) We used limiting dilution to select single-cell reporter clones (A6-G3), expanded them, and characterized them to determine the number of reporter integrations at the *AAVS1* locus on chromosome 19 which exists in 3 copies in HEK293T cells. Average Citrine fluorescence intensity for the single clones as measured by flow cytometry (top). Genomic PCR results with primers that detected integrated reporter or the WT sequences without integration (bottom). (**B**) Estimated copy number of the reporter gene relative to the Albumin gene measured by droplet digital PCR. (**C**) The percentage of chromosomes with positive DNA FISH readout signal at the reporter out of all chromosomes 19 detected by the fiducial signal. (**D**) Histograms of the reporter Citrine fluorescence after 5 days of KRAB recruitment and 16 days passage after dox removal using the original B9 clone (left), and using the reactivated cells after sorting (right). No dox control distribution is shown in gray. Two replicates for each condition were superimposed. (**E**) Histograms of the reporter Citrine fluorescence intensity of the B9 line after 5 days with dox and 12 days of dox removal (blue) versus no dox (yellow) as measured by flow cytometry. Note that reactivated cells show lower Citrine expression levels compared to no dox, which is not observed in the single integrant A6 clone in (F). (**F**) Histograms of the reporter Citrine fluorescence intensity of the A6 line after 5 days with dox and 12 days of dox removal (blue) versus no dox (yellow) as measured by flow cytometry. (**G**) Histograms of Citrine fluorescence for the double reporter B9-KRAB cell line were fitted with a normal distribution (imposing a ± 3 sigma cutoff) for cells with no dox (top) or treated with dox for 5 days (bottom). (**H**) Fluorescence intensity distribution of the Citrine reporter after reactivation in the double reporter B9-KRAB cell line (blue) was fitted with a probability distribution (red) that assumes stochasticity happens at the cell level (i.e. if a given

reporter activates in a cell, the other reporter also reactivates in that cell, as shown in the schematic on the bottom). This cell stochasticity predicts a higher fluorescence distribution (red) than measured (blue). (**I**) Fluorescence intensity distribution of the Citrine reporter after reactivation in the double reporter B9-KRAB cell line (blue) was fitted with probability distribution assuming stochasticity happens at the individual locus level (i.e. in a given cell, each integrated reporter can activate independently of the reporter integrated on another chromosome, as shown in the schematic on the bottom). Note that the independent locus stochasticity model in (I) shows better fitting than the cell-driven stochasticity model in (H).

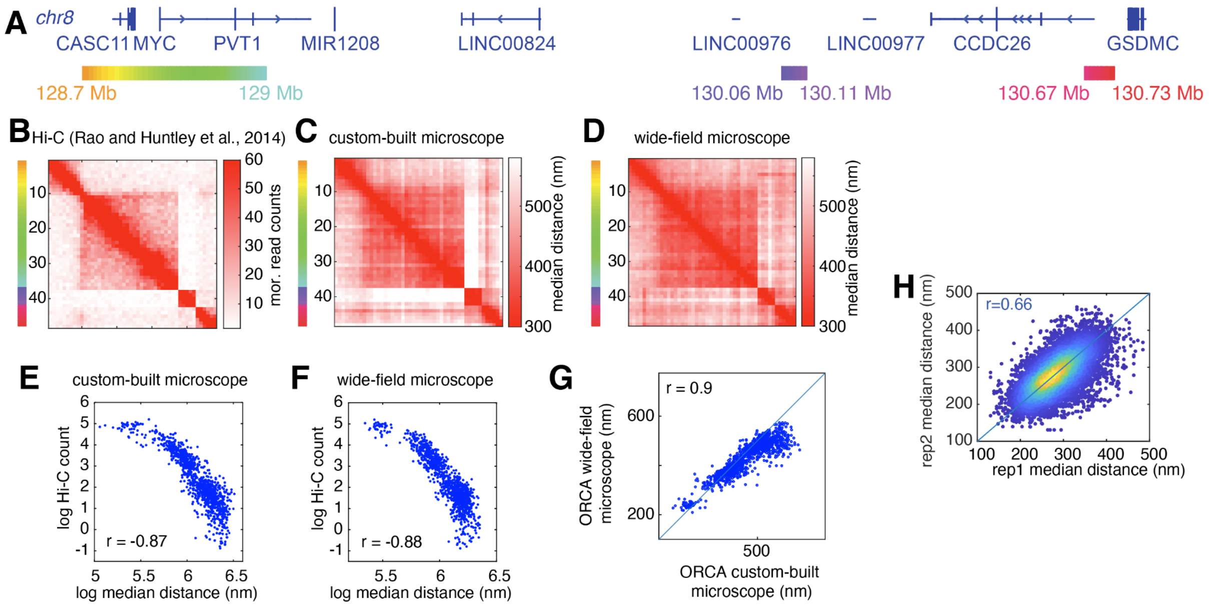

Fig. S2. Validation of the ORCA system built on a wide-field microscope.

(**A**) ORCA probe design at the MYC locus. Gene annotations (top). Locations of ORCA probes are shown in different colors (bottom). Each probe set targets a 10 kb segment. (**B**) A heatmap showing the pairwise contact frequency as computed from bulk Hi-C normalized reads at the *MYC* locus (data from Rao SSP and Huntley MH *et al.*, *Cell*, 2014(*29*)). (**C**) Median distance map between pairs of segments across the *MYC* locus measured using ORCA on a custom-built microscope that has been used for previous publications (Boettiger lab(*26*)). (**D**) Median distance map of ORCA on the wide-field microscope used in this study (Bintu lab). (**E**) Correlation between Hi-C normalized reads and ORCA median distances for the MYC locus measured on the custom-built microscope. (**F**) Correlation between Hi-C normalized reads and ORCA median distances measured on the wide-field microscope. Linear correlation between Hi-C number of reads and median distances in a log scale was observed as reported before(*80*). (**G**) Correlation between median distances measured by ORCA on a custom-built microscope vs. the wide-field microscope used in this study. (**H**) Median distances of all pairwise segments from all pairs of replicates across all datasets from all HEK293T cell lines derivatives of the B9 clone reported in this study are superimposed. In the case of multiple replicates, all combinations of replicate pairs are all plotted (eg. rep1/rep2, rep2/rep3, rep1/rep3, etc.). The overall correlation coefficient is 0.66.

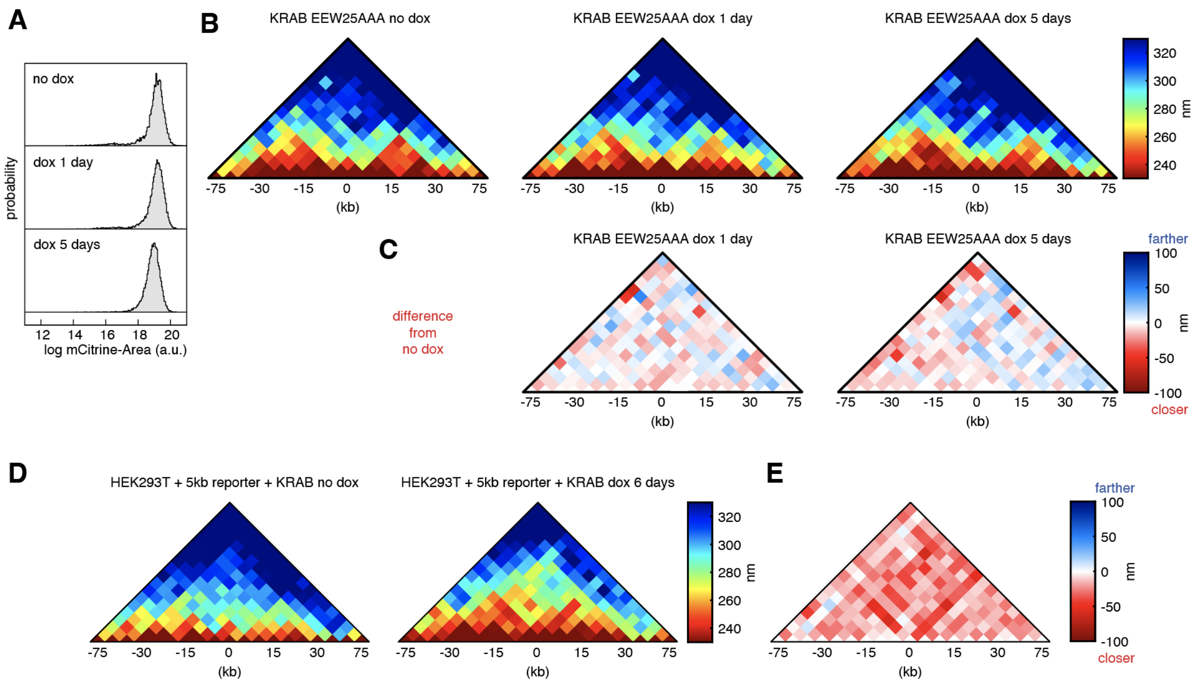

Fig. S3. Control experiments for KRAB-mediated chromatin compaction.

(**A**) Flow cytometry histograms of the reporter Citrine fluorescence intensity of the double integrant clone (B9) with no dox, dox 1 day, or dox 5 days. (**B**) Median distance maps from chromatin traces upon KRAB EEW25AAA mutant recruitment for 1 day or 5 days and no dox control. (**C**) Subtracted median distance maps upon 1 day or 5 days KRAB EEW25AAA recruitment compared to the no dox control. Two replicates are averaged. (**D**) Median pairwise distance maps from chromatin traces in polyclonal HEK293T cells harboring Citrine and mCherry reporters separated by a 5-kb spacer(*31*), measured upon KRAB mutant recruitment for 6 days (right) and no dox control (left). (**E**) Subtracted median distance maps upon 6 days KRAB recruitment compared to the no dox control using polyclonal HEK293T cells harboring Citrine and mCherry reporters with a 5-kb spacer. Two replicates are averaged.

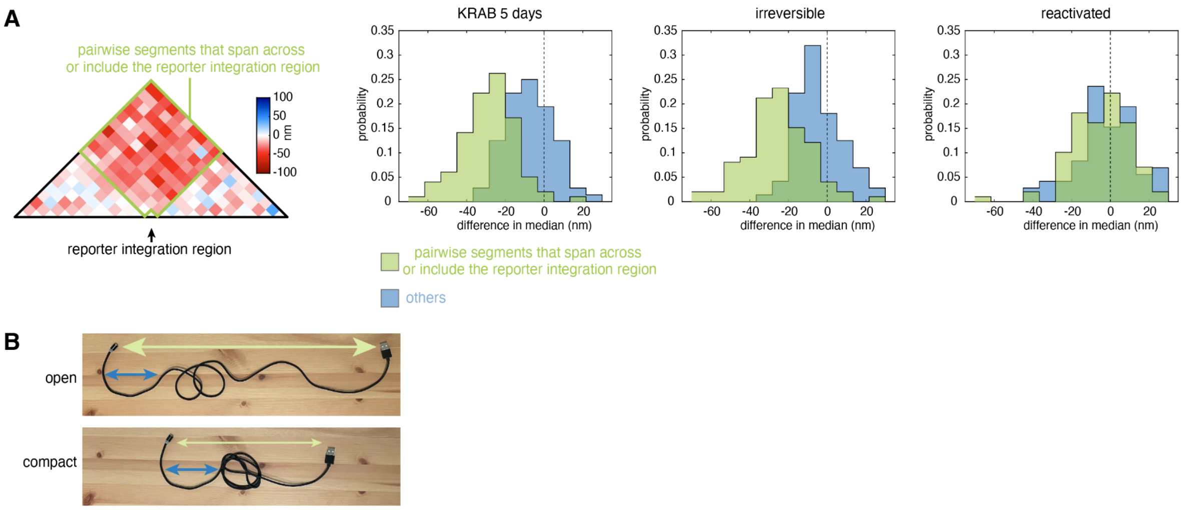

Fig. S4. Localized chromatin compaction.

(**A**) A green box highlights pairwise segments (left) that span across the reporter integration region (contain the reporter site between them). Histograms of subtracted median distances between segments (right) that span across or include the reporter integration region (green box in the left figure), and other pairwise segments (blue). (**B**) Visual interpretation of locally compacted chromatin. "Compaction" at the center of a cord makes end-to-end distance shorter (yellow arrows), without significant change in the distance between pairs outside the compacted region (blue arrows).

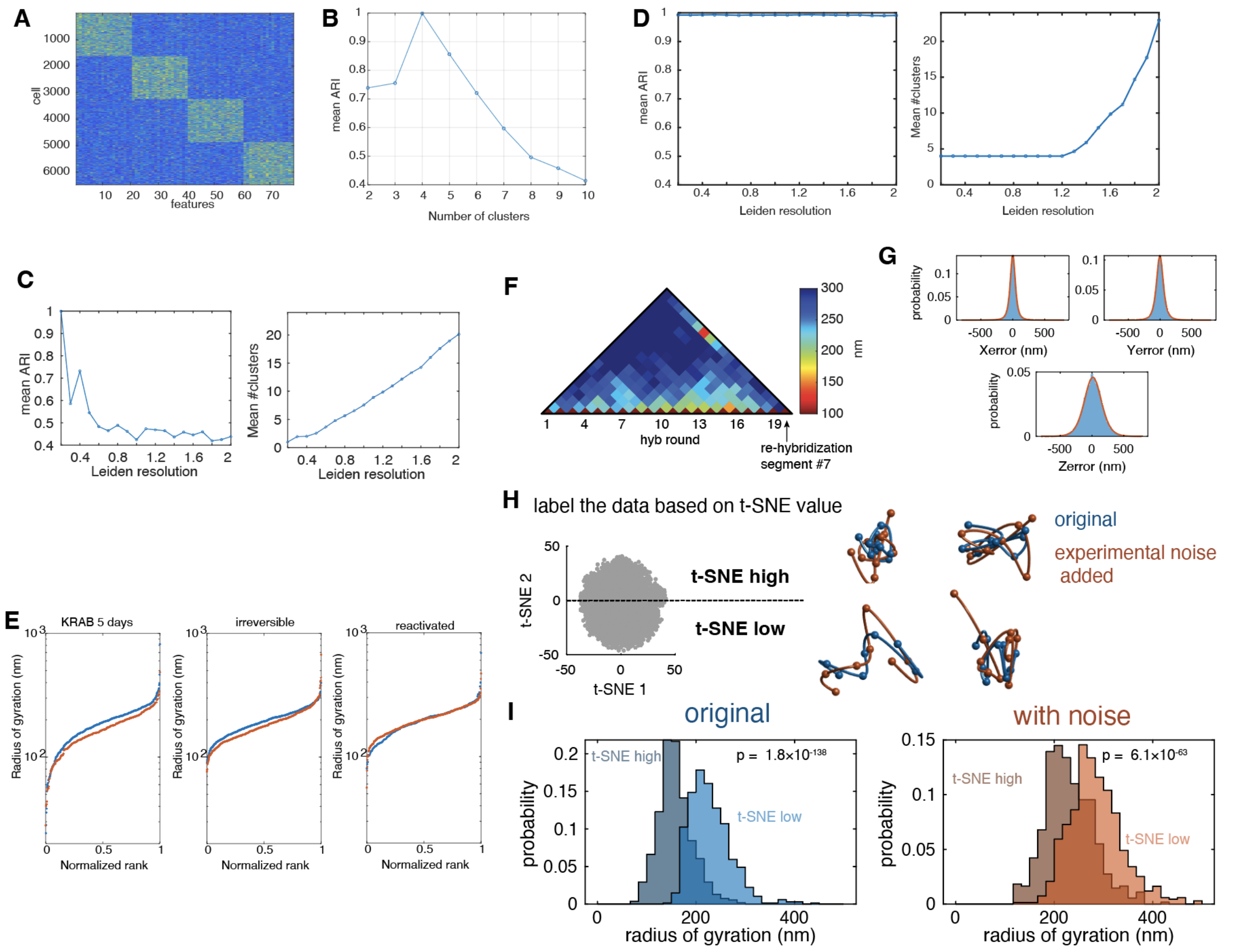

Fig. S5. The effect of experimental noise on heterogeneity in the t-SNE dimensionality reduction analysis.

(**A**) Synthetic dataset, including four distinct clusters and added noise, used to validate the clustering algorithm. (**B**) Mean Adjusted Rand Index (ARI) for a K-means clustering parameter sweep, with the number of clusters as the parameter. The maximum mean ARI at K=4 indicates that four groups represent the most reproducible clustering, accurately confirming the inherent number of clusters in the dataset. (**C** and **D**) Mean Adjusted Rand Index (left) and mean number of clusters (right) resulting from a parameter sweep of the Leiden clustering resolution using the chromatin tracing data (C) analyzed in Fig. 2 or the synthetic dataset (D) presented in (A). Note that (D) shows high reproducibility and four clusters at low resolution, which matches the nature of the data. (**E**) Representative rank plots for the radius of gyration of single-cell traces for 5 days KRAB recruitment, irreversibly silenced, or reactivated conditions in red, and no dox control in blue. (**F**) A median distance map for B9-KRAB no dox cells with segment #7 imaged during round 7 of imaging and re-hybridized at the end of chromatin tracing (black arrow). Note that the distance between segment #7 and the re-hybridized segment #7 is the smallest across all pairwise distances (red). (**G**) The difference between 1st and 2nd hybridization of segment #7 in each direction in 3D (blue). The distributions were fitted with a t-location scale distribution (red). (**H**) Samples were classified into t-SNE high (t-SNE 2 > 0) or t-SNE low (t-SNE 2 < 0) (left). Simulated experimental noise was sampled from the fitted distribution in (G), then added to each chromatin trace (original

traces are in blue and traces with simulated experimental noise are in red) (right). (**I**) The distribution of radius of gyration for the t-SNE low group (dark color) and the t-SNE high group (light color) before (blue) or after (red) adding experimental noise. The p-values were calculated using the Wilcoxon Rank Sum test.

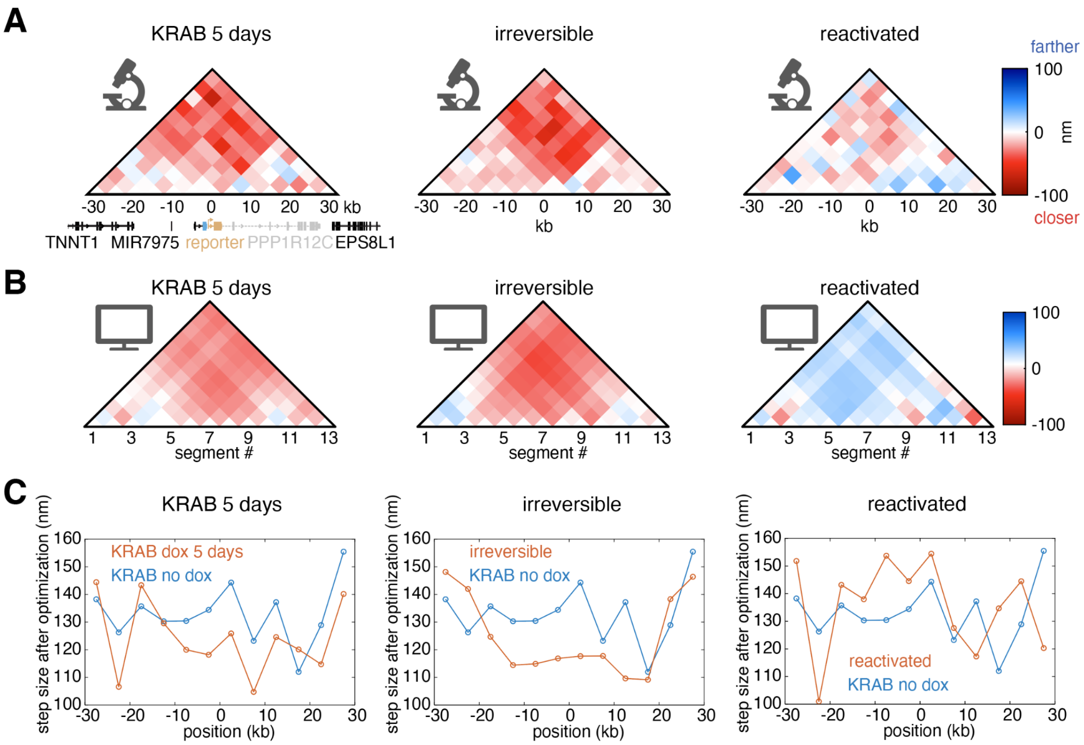

Fig. S6. Bayesian optimization to fit a locally-compacted 3D random-walk polymer model to experimental data.

(**A**) Subtracted median distance maps from the experimental data used for fitting. (**B**) Subtracted median distance maps generated using the fitted random-walk polymer model. (**C**) Fitted step sizes at each genomic position.

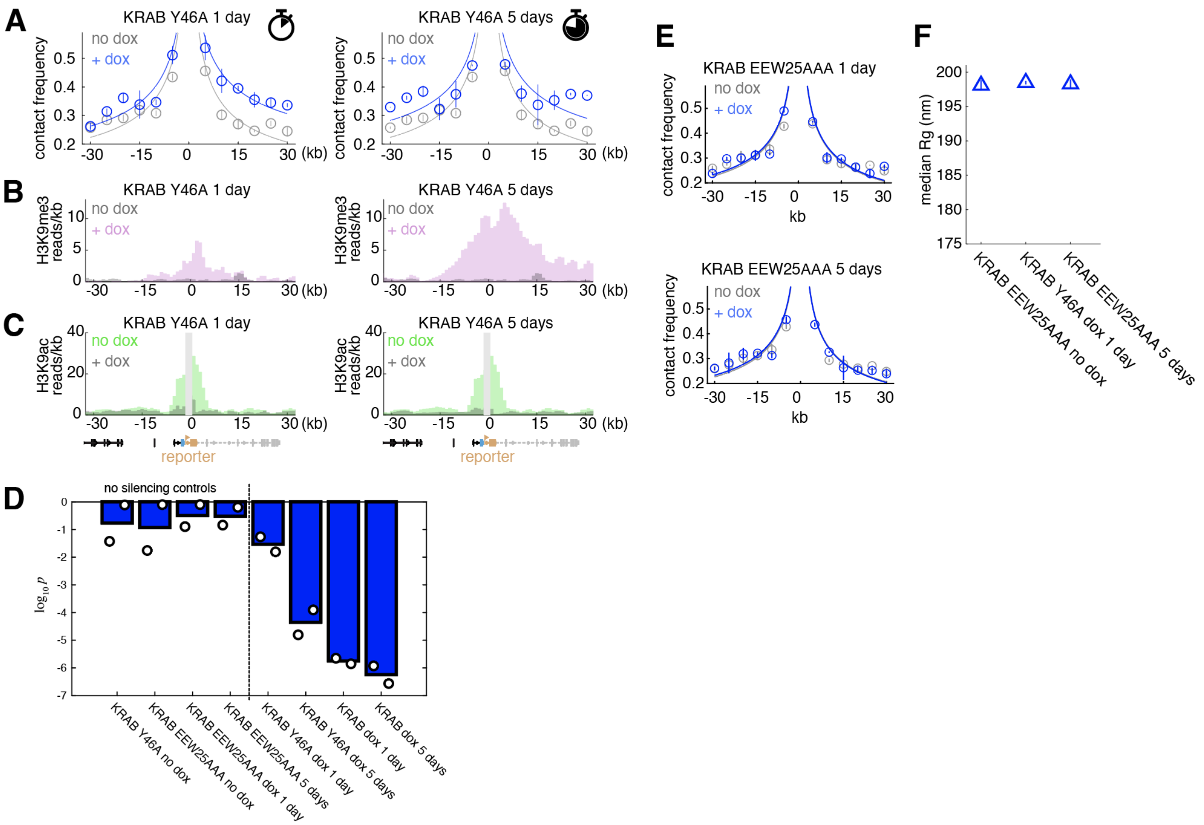

Fig. S7. KRAB mutant recruitment and statistical analysis of the medians radii of gyration.

(**A**) Contact frequency between the reporter integrated region and other genomic regions measured by ORCA at the end of 1 day (left) or 5 days (right) of KRAB Y46A recruitment (blue) and in the no dox control condition (gray). Lines show power-law decay fitting results. Circles show the average, with vertical and horizontal bars indicating the standard deviation. (**B**) Genome traces showing normalized number of reads (averaged from two replicates) after CUT&RUN against H3K9me3 (magenta) as a function of distance around the reporter integration site (at 0 kb) after 1 day (left) or 5 days (right) of KRAB Y46A recruitment, and in no dox control (gray). The traces are shown in 1-kb binning with the moving average over 3-kb windows. (**C**) Genome traces showing normalized number of reads (averaged from two replicates) after CUT&RUN against H3K9ac (gray) as a function of distance around the reporter integration site (at 0 kb) after 1 day (left) or 5 days (right) of KRAB Y46A recruitment, and in no dox control (green). The traces are shown in 1-kb binning with the moving average over 3-kb windows. (**D**) Wilcoxon Rank Sum test was carried out as a measure of the difference in the median radius of gyration for each condition compared to WT KRAB no dox cells. Bar plots show the average. Dots represent two biological replicates. (**E**) Contact frequency between the reporter integrated region and other genomic regions measured by ORCA at the end of 1 day (top) or 5 days (bottom) of KRAB EEW25AAA recruitment (blue) and in the no dox control condition (gray). Lines show power-law decay fitting results. Circles show the average, with vertical and horizontal bars indicating the standard deviation. (**F**) The median radius of gyration for the -30 to 30 kb region around the reporter at the end of KRAB EEW25AAA recruitment. Triangles show the average, with vertical bars indicating the standard deviation.

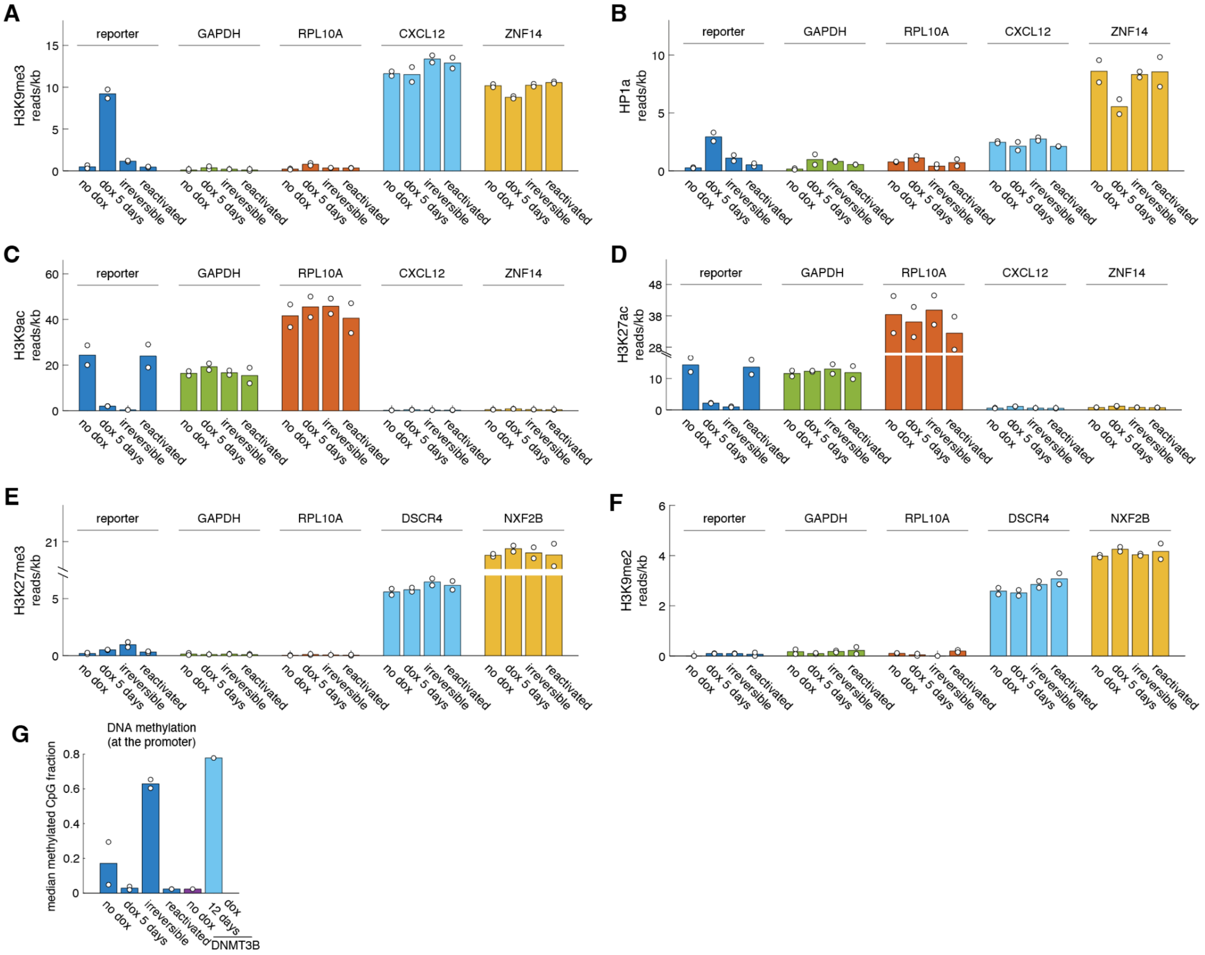

Fig. S8. Changes in epigenetic modifications upon KRAB recruitment.

(**A** and **B**) Quantification of the normalized H3K9me3 (A) or HP1α (B) CUT&RUN reads at the integrated reporter region, and negative (GAPDH, RPL10A) and positive (CXCL12, ZNF14) control gene loci. (**C** and **D**) Quantification of the normalized H3K9ac (C) or H3K27ac (D) CUT&RUN reads at the integrated reporter region, positive (GAPDH, RPL10A) and negative (CXCL12, ZNF14) control gene loci. Reads aligned to the human EF1alpha promoter were excluded (see Materials and Methods). (**E** and **F**) Quantification of the normalized H3K27me3 (E) or H3K9me2 (F) CUT&RUN reads at the integrated reporter region, negative (GAPDH, RPL10A) and positive (DSCR4, NXF2B) control gene loci. Bars show the average from two replicates, dots represent individual replicates. All CUT&RUN experiments were done with the B9 clone. Note that epigenetic modification enrichment at positive and negative control genes are consistent across conditions. (**G**) Quantification of the methylated CpG fraction at the pEFalpha promoter using EM-Seq.

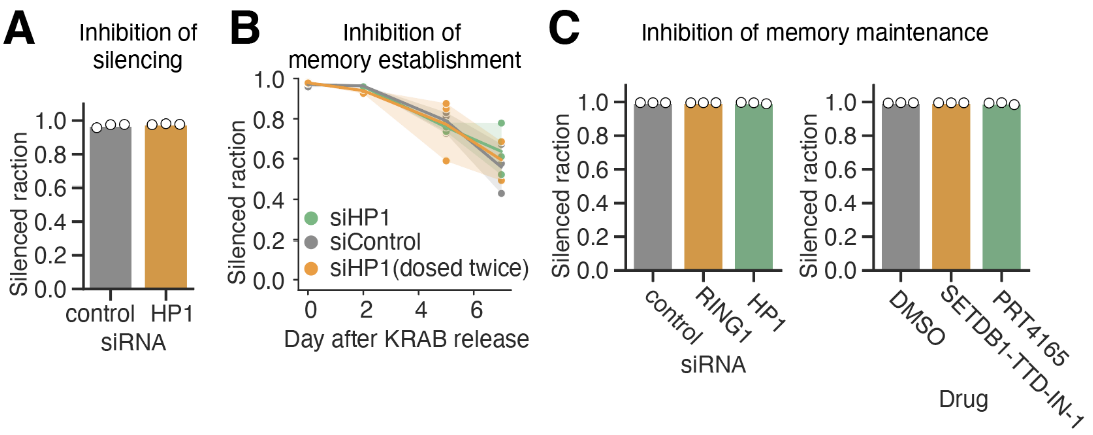

Fig. S9. HP1 and PRC1 inhibitors do not impair KRAB-mediated gene silencing and memory formation.

(**A**) Fraction of cells with Citrine silenced after 5 days of KRAB recruitment at saturating dox concurrent with siRNA transfections using either a mixture of siRNAs targeting HP1alpha, HP1beta, and HP1gamma (orange) or scramble siRNA control (gray). The silenced fraction is normalized to the no dox control for each condition. Bars show the average of three replicates, circles show each replicate. See Table S4 for concentrations. (**B**) Time-course showing the fraction of cells with Citrine remaining silenced during the KRAB release period (after 5 days of recruitment) upon the addition of siRNA mixture against HP1s either at the start of the release period (green) or dosed twice: at the start of the release period and at day 5 in the release period (orange). The silenced fraction is normalized to the no dox control for each condition. Lines show the average of three replicates, circles show each replicate. See Table S4 for concentrations. (**C**) Fraction of cells with Citrine silenced after 4 days of siRNA (left) or inhibitor (right) treatment on irreversibly silenced cells. SETDB1-TTD-IN-1 is a SETDB1 inhibitor; PRT4165 is a PRC1 inhibitor. Bars show the average of three replicates, circles show each replicate. See Table S4 for siRNAs and inhibitors catalog numbers and concentrations.

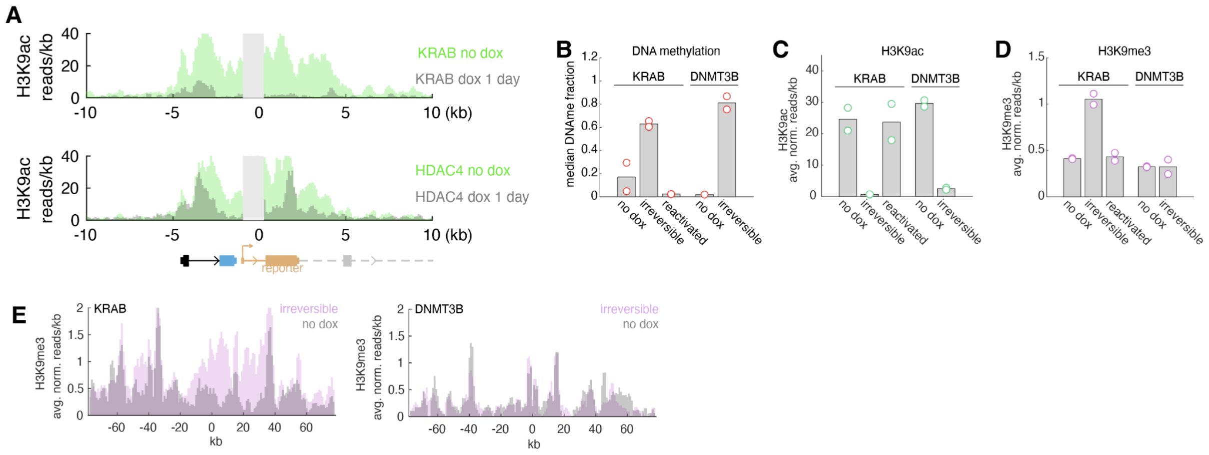

Fig. S10. Molecular profiling upon HDAC4 and DNMT3B recruitment.

(**A**) Genome traces showing the normalized number of reads after CUT&RUN against H3K9ac in KRAB or HDAC4 recruitment for 1 day (gray). No dox control is shown in green. The signal is binned into 100-bp windows with the moving average in 300-bp windows. (**B**) Median DNA methylation density in amplicon in memory phase after KRAB or DNMT3B recruitment. Bars show the average of two replicates, circles show each replicate. (**C**) H3K9ac CUT&RUN signal integrated over the reporter region in memory phase after KRAB or DNMT3B recruitment. Bars show the average of two replicates, circles show each replicate. Reads aligned to the human EF1alpha promoter were excluded (see Materials and Methods). (**D**) H3K9me3 CUT&RUN signal integrated over -20:40 kb around the reporter in memory phase after KRAB or DNMT3B recruitment. Bars show the average of two replicates, circles show each replicate. (**E**) Genome traces showing normalized number of reads after CUT&RUN against H3K9me3 as a function of distance around the reporter integration site (at 0 kb) in irreversibly silenced cells (purple) after KRAB (left) or DNMT3B (right) recruitment, and in no dox control (gray). The traces are shown in 1-kb binning with the moving average over 3-kb windows.

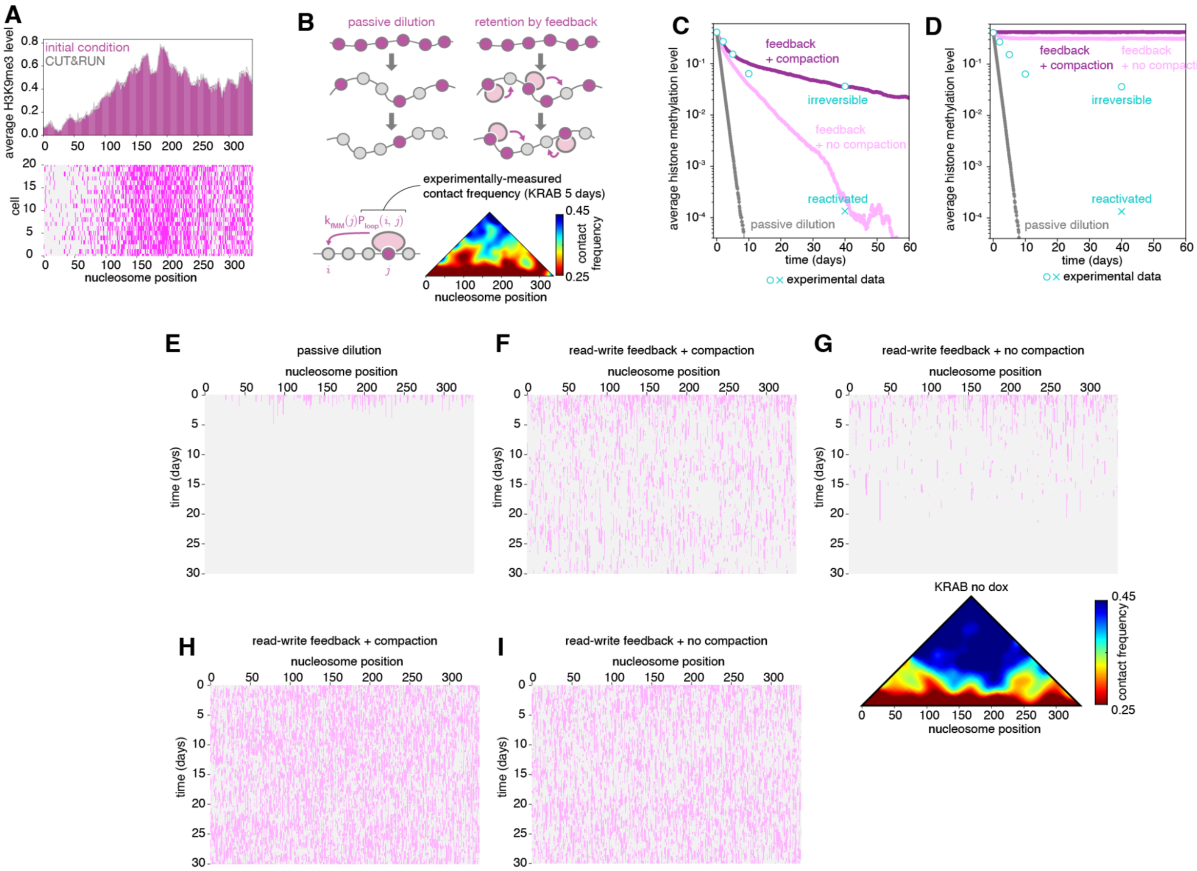

Fig. S11. The positive feedback model reproduces slow H3K9me3 decay.

(**A**) Interpolated CUT&RUN genome trace of H3K9me3 (in this case measured at the end of 5 days of KRAB recruitment at 1000ng/ml dox) shown in gray with the fraction of methylated nucleosomes in the initial condition of the simulation generated so that it matches the experiment result shown in purple (top). 20 representative initial conditions (bottom). (**B**) Schematic representing two different scenarios of H3K9me3 decay dynamics: only passive dilution (upper left) or positive feedback reaction mediated by reader-writer module to retain H3K9me3 (upper right). Experimentally-derived looping probability between nucleosomes (bottom). The contact frequency matrix derived from ORCA experiments is linearly interpolated (see Materials and Methods). (**C**) Time-course showing average methylation level from the simulation, where methylation level is defined as the fraction of nucleosomes with methylated state in the system. (**D**) Same as (C), using simulation results with higher feedback strength (k_{fMM} in A). (**E** to **G**) Representative dynamics of the simulation with only passive dilution (E), positive feedback reaction (F), positive feedback reaction but no compaction (G). The contact frequency matrix used for no compaction simulation is also shown at the bottom of (G). (**H** and **I**) Representative dynamics of the simulation with a high feedback reaction rate (k_{fMM} in A) with positive feedback reaction (H), positive feedback reaction but no compaction (I). Note that a higher feedback reaction rate (k_{fMM} in A) leads to strongly sustained H3K9me3 independent of contact frequency, but does not match the experimentally measured H3K9me3 decay.

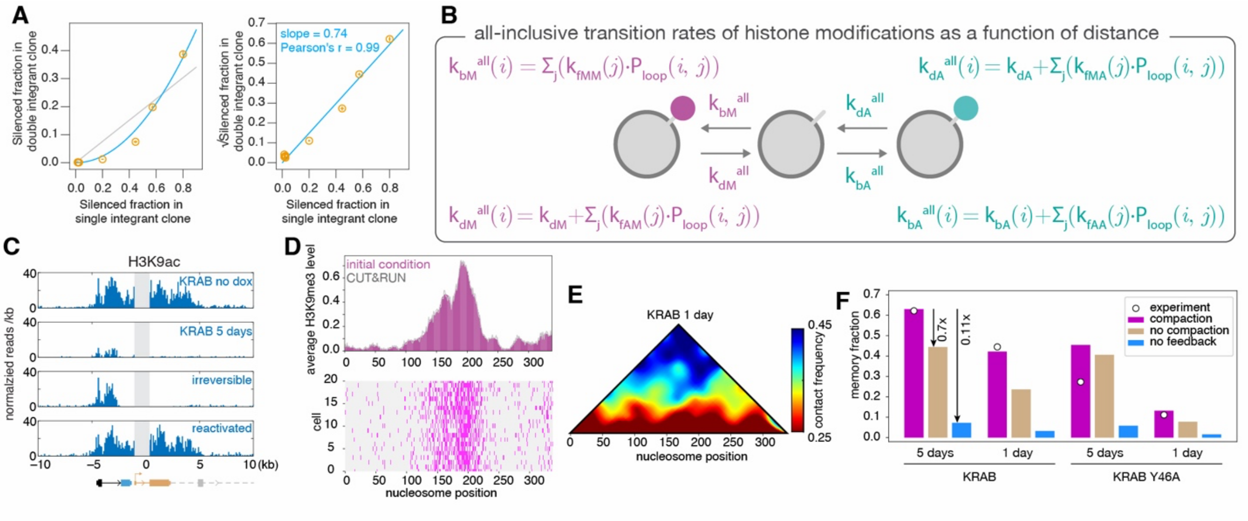

Fig. S12. Methylation-acetylation spreading model.

(**A**) Memory fraction of cells in the double integrant clone is plotted against that of the single integrant clone. Conditions are the same as in Fig. 3B. The memory fraction of cells is better fitted with a quadratic function shown in blue than a linear function shown in gray (left). The square root of the memory fraction of cells in the double integrant clone shows linear correlation against the memory fraction in the single integrant clone (right). The slope of linear fitting to the square root of memory in the double integrant clone and the memory in the single integrant clone is not equal to 1 (around 0.74), suggesting that the double integrant clone is less efficient in memory formation than the single integrant clone. (**B**) All-inclusive transition rates between methylated, unmodified and acetylated states. (**C**) Genome traces showing the normalized number of reads (averaged from two replicates) after CUT&RUN against H3K9ac under no dox, 5 days of KRAB recruitment, irreversibly silenced, or reactivated conditions. The human EF1alpha promoter driving the reporter gene is masked with gray rectangles since sequencing reads also align to the endogenous copies of EF1alpha that are not targeted with KRAB and are therefore expected to retain acetylation. (**D**) Initial condition of histone methylation used for 1 day of KRAB recruitment simulation. (**E**) Interpolated contact frequency matrix used for 1 day of KRAB recruitment simulation. (**F**) Fraction of irreversibly silenced loci from the simulation with different initial conditions and looping probability, as well as different feedback scenarios. Dots show experimentally-derived silenced fraction of loci for each condition.

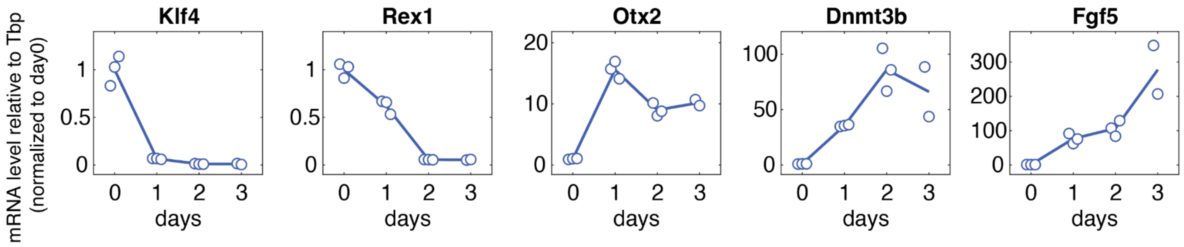

Fig. S13. Gene expression changes for cell markers upon mouse ES cell differentiation.

Marker genes for the pluripotent state (*Klf4*, *Rex1*) and the differentiated state (*Otx2*, *Dnmt3b*, *Fgf5*) were quantified by RT-qPCR. The mRNA levels were quantified relative to Tbp, then normalized to the average mRNA level at day 0. Line plots show the average across two or three biological replicates.

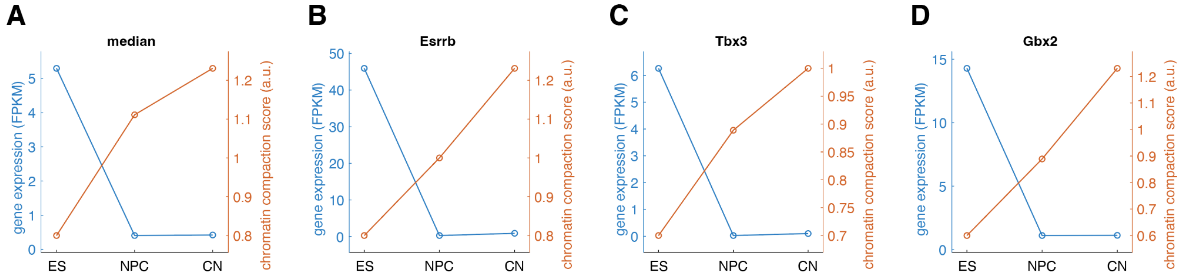

Fig. S14. Analysis of Hi-C and RNA-seq data during mouse ES cell differentiation.

(**A**) The median gene expression level in FPKM (blue) and the median chromatin compaction score (orange) of the 97 selected genes that show fast silencing followed by slower compaction (see Materials and Methods, ref. *(44)*) as they progress during the stages of differentiation: ES, NPC, and CN. (**B to D)** Gene expression level (blue) and chromatin compaction score (orange) of example genes encoding for pluripotency-related transcription factors *Esrrb* (B), *Tbx3* (C), and *Gbx2* (D) at the ES, NPC, and CN stage.

Table S1. Summary table of the number of replicates and single-cell chromatin traces for each experimental condition.

Table S2. Parameter values for stochastic simulations of chromatin modifications dynamics during memory establishment. The rates are listed for the histone methylation-acetylation feedback model presented in Fig. 6 (column 4) and the simplified positive feedback model with only histone methylation presented in Fig. S11 (column 3). N.A. indicates rates that are not applicable in the simplified model. See Fig. S12B for all-inclusive transition rates calculated using these parameters and the probability of looping as a function of distance.

Table S3. List of inhibitors and siRNAs catalog numbers, concentrations, and treatments conditions.

Data S1. A list of 97 genes showing rapid silencing and gradual chromatin compaction during mouse ES cell differentiation into cortical neurons identified by our analysis.

REFERENCES

1. J. S. Becker, D. Nicetto, K. S. Zaret, H3K9me3-dependent heterochromatin: Barrier to cell fate changes. *Trends Genet.* **32**, 29–41 (2016).

2. S. Sun, L. B. Barreiro, The epigenetically-encoded memory of the innate immune system. *Curr. Opin. Immunol.* **65**, 7–13 (2020).

3. Z. Chen, R. Natarajan, Epigenetic modifications in metabolic memory: What are the memories, and can we erase them? *Am. J. Physiol. Cell Physiol.* **323**, C570–C582 (2022).

4. L. Kiefer, S. Gaudin, S. M. Rajkumar, G. I. F. Servito, J. Langen, M. H. Mui, S. Nawsheen, D. Canzio, Tuning cohesin trajectories enables differential readout of the Pcdhα cluster across neurons. *Science* **385**, eadm9802 (2024).

5. S. Thiagalingam, Epigenetic memory in development and disease: Unraveling the mechanism. *Biochim. Biophys. Acta Rev. Cancer* **1873**, 188349 (2020).

6. J. Xu, Y. Liu, Probing chromatin compaction and its epigenetic states in situ with single-molecule localization-based super-resolution microscopy. *Front. Cell Dev. Biol.* **9**, 653077 (2021).

7. B. van Steensel, A. S. Belmont, Lamina-associated domains: Links with chromosome architecture, heterochromatin, and gene repression. *Cell* **169**, 780–791 (2017).

8. J. F. Margolin, J. R. Friedman, W. K. Meyer, H. Vissing, H. J. Thiesen, F. J. Rauscher III, Krüppel-associated boxes are potent transcriptional repression domains. *Proc. Natl. Acad. Sci. U.S.A.* **91**, 4509–4513 (1994).

9. L. A. Gilbert, M. H. Larson, L. Morsut, Z. Liu, G. A. Brar, S. E. Torres, N. Stern-Ginossar, O. Brandman, E. H. Whitehead, J. A. Doudna, W. A. Lim, J. S. Weissman, L. S. Qi, CRISPR-mediated modular RNA-guided regulation of transcription in eukaryotes. *Cell* **154**, 442–451 (2013).

10. H. Peng, L. C. Gibson, A. D. Capili, K. L. B. Borden, M. J. Osborne, S. L. Harper, D. W. Speicher, K. Zhao, R. Marmorstein, T. A. Rock, F. J. Rauscher III, The structurally disordered KRAB repression domain is incorporated into a protease resistant core upon binding to KAP-1-RBCC domain. *J. Mol. Biol.* **370**, 269–289 (2007).

11. D. C. Schultz, K. Ayyanathan, D. Negorev, G. G. Maul, F. J. Rauscher III, SETDB1: A novel KAP-1-associated histone H3, lysine 9-specific methyltransferase that contributes to HP1-mediated silencing of euchromatic genes by KRAB zinc-finger proteins. *Genes Dev.* **16**, 919–932 (2002).

12. K. Ayyanathan, M. S. Lechner, P. Bell, G. G. Maul, D. C. Schultz, Y. Yamada, K. Tanaka, K. Torigoe, F. J. Rauscher III, Regulated recruitment of HP1 to a euchromatic gene induces mitotically heritable, epigenetic gene silencing: A mammalian cell culture model of gene variegation. *Genes Dev.* **17**, 1855–1869 (2003).

13. N. A. Hathaway, O. Bell, C. Hodges, E. L. Miller, D. S. Neel, G. R. Crabtree, Dynamics and memory of heterochromatin in living cells. *Cell* **149**, 1447–1460 (2012).

14. Y. Feng, Y. Wang, X. Wang, X. He, C. Yang, A. Naseri, T. Pederson, J. Zheng, S. Zhang, X. Xiao, W. Xie, H. Ma, Simultaneous epigenetic perturbation and genome imaging reveal distinct roles of H3K9me3 in chromatin architecture and transcription. *Genome Biol.* **21**, 296 (2020).

15. D. Nicetto, K. S. Zaret, Role of H3K9me3 heterochromatin in cell identity establishment and maintenance. *Curr. Opin. Genet. Dev.* **55**, 1–10 (2019).

16. T. Zhang, S. Cooper, N. Brockdorff, The interplay of histone modifications—Writers that read. *EMBO Rep.* **16**, 1467–1481 (2015).

17. M. M. Müller, B. Fierz, L. Bittova, G. Liszczak, T. W. Muir, A two-state activation mechanism controls the histone methyltransferase Suv39h1. *Nat. Chem. Biol.* **12**, 188–193 (2016).

18. R. Margueron, N. Justin, K. Ohno, M. L. Sharpe, J. Son, W. J. Drury III, P. Voigt, S. R. Martin, W. R. Taylor, V. De Marco, V. Pirrotta, D. Reinberg, S. J. Gamblin, Role of the polycomb protein EED in the propagation of repressive histone marks. *Nature* **461**, 762–767 (2009).

19. A. V. Probst, E. Dunleavy, G. Almouzni, Epigenetic inheritance during the cell cycle. *Nat. Rev. Mol. Cell Biol.* **10**, 192–206 (2009).

20. H. J. Muller, Types of visible variations induced by x-rays in Drosophila. *J. Genet.* **22**, 299–334 (1930).

21. J. F. Nickels, A. K. Edwards, S. J. Charlton, A. M. Mortensen, S. C. L. Hougaard, A. Trusina, K. Sneppen, G. Thon, Establishment of heterochromatin in domain-size-dependent bursts. *Proc. Natl. Acad. Sci. U.S.A.* **118**, e2022887118 (2021).

22. L. Bintu, J. Yong, Y. E. Antebi, K. McCue, Y. Kazuki, N. Uno, M. Oshimura, M. B. Elowitz, Dynamics of epigenetic regulation at the single-cell level. *Science* **351**, 720–724 (2016).

23. A. Amabile, A. Migliara, P. Capasso, M. Biffi, D. Cittaro, L. Naldini, A. Lombardo, Inheritable silencing of endogenous genes by hit-and-run targeted epigenetic editing. *Cell* **167**, 219–232.e14 (2016).

24. M. V. Van, T. Fujimori, L. Bintu, Nanobody-mediated control of gene expression and epigenetic memory. *Nat. Commun.* **12**, 537 (2021).

25. P. J. Skene, S. Henikoff, An efficient targeted nuclease strategy for high-resolution mapping of DNA binding sites. *eLife* **6**, e21856 (2017).

26. L. J. Mateo, S. E. Murphy, A. Hafner, I. S. Cinquini, C. A. Walker, A. N. Boettiger, Visualizing DNA folding and RNA in embryos at single-cell resolution. *Nature* **568**, 49–54 (2019).

27. J. Tycko, N. DelRosso, G. T. Hess, Aradhana, A. Banerjee, A. Mukund, M. V. Van, B. K. Ego, D. Yao, K. Spees, P. Suzuki, G. K. Marinov, A. Kundaje, M. C. Bassik, L. Bintu, High-

throughput discovery and characterization of human transcriptional effectors. *Cell* **183**, 2020–2035.e16 (2020).

28. I. J. Roney, A. D. Rudner, J.-F. Couture, M. Kærn, Improvement of the reverse tetracycline transactivator by single amino acid substitutions that reduce leaky target gene expression to undetectable levels. *Sci. Rep.* **6**, 27697 (2016).

29. S. S. P. Rao, M. H. Huntley, N. C. Durand, E. K. Stamenova, I. D. Bochkov, J. T. Robinson, A. L. Sanborn, I. Machol, A. D. Omer, E. S. Lander, E. L. Aiden, A 3D map of the human genome at kilobase resolution reveals principles of chromatin looping. *Cell* **159**, 1665–1680 (2014).

30. L. J. Mateo, N. Sinnott-Armstrong, A. N. Boettiger, Tracing DNA paths and RNA profiles in cultured cells and tissues with ORCA. *Nat. Protoc.* **16**, 1647–1713 (2021).

31. S. Lensch, M. H. Herschl, C. H. Ludwig, J. Sinha, M. M. Hinks, A. Mukund, T. Fujimori, L. Bintu, Dynamic spreading of chromatin-mediated gene silencing and reactivation between neighboring genes in single cells. *eLife* **11**, e75115 (2022).

32. A. Hafner, M. Park, S. E. Berger, S. E. Murphy, E. P. Nora, A. N. Boettiger, Loop stacking organizes genome folding from TADs to chromosomes. *Mol. Cell* **83**, 1377–1392.e6 (2023).

33. L.-F. Chen, H. K. Long, M. Park, T. Swigut, A. N. Boettiger, J. Wysocka, Structural elements promote architectural stripe formation and facilitate ultra-long-range gene regulation at a human disease locus. *Mol. Cell* **83**, 1446–1461.e6 (2023).

34. J. R. Friedman, W. J. Fredericks, D. E. Jensen, D. W. Speicher, X. P. Huang, E. G. Neilson, F. J. Rauscher III, KAP-1, a novel corepressor for the highly conserved KRAB repression domain. *Genes Dev.* **10**, 2067–2078 (1996).

35. W. Zeng, A. R. Ball Jr., K. Yokomori, HP1: heterochromatin binding proteins working the genome. *Epigenetics* **5**, 287–292 (2010).

36. Z. Wang, G. Qin, T. C. Zhao, HDAC4: mechanism of regulation and biological functions. *Epigenomics* **6**, 139–150 (2014).

37. I. B. Dodd, M. A. Micheelsen, K. Sneppen, G. Thon, Theoretical analysis of epigenetic cell memory by nucleosome modification. *Cell* **129**, 813–822 (2007).

38. F. Erdel, E. C. Greene, Generalized nucleation and looping model for epigenetic memory of histone modifications. *Proc. Natl. Acad. Sci. U.S.A.* **113**, E4180–E4189 (2016).

39. A. R. Cutter DiPiazza, N. Taneja, J. Dhakshnamoorthy, D. Wheeler, S. Holla, S. I. S. Grewal, Spreading and epigenetic inheritance of heterochromatin require a critical density of histone H3 lysine 9 tri-methylation. *Proc. Natl. Acad. Sci. U.S.A.* **118**, e2100699118 (2021).

40. J. A. Owen, D. Osmanović, L. Mirny, Design principles of 3D epigenetic memory systems. *Science* **382**, eadg3053 (2023).

41. A. Movilla Miangolarra, D. S. Saxton, Z. Yan, J. Rine, M. Howard, Two-way feedback between chromatin compaction and histone modification state explains Saccharomyces cerevisiae heterochromatin bistability. *Proc. Natl. Acad. Sci. U.S.A.* **121**, e2403316121 (2024).

42. A. Dubois, L. Vincenti, A. Chervova, M. V. C. Greenberg, S. Vandormael-Pournin, D. Bourc'his, M. Cohen-Tannoudji, P. Navarro, H3K9 tri-methylation at Nanog times differentiation commitment and enables the acquisition of primitive endoderm fate. *Development* **149**, dev201074 (2022).

43. T. Kalkan, N. Olova, M. Roode, C. Mulas, H. J. Lee, I. Nett, H. Marks, R. Walker, H. G. Stunnenberg, K. S. Lilley, J. Nichols, W. Reik, P. Bertone, A. Smith, Tracking the embryonic stem cell transition from ground state pluripotency. *Development* **144**, 1221–1234 (2017).

44. B. Bonev, N. Mendelson Cohen, Q. Szabo, L. Fritsch, G. L. Papadopoulos, Y. Lubling, X. Xu, X. Lv, J.-P. Hugnot, A. Tanay, G. Cavalli, Multiscale 3D genome rewiring during mouse neural development. *Cell* **171**, 557–572.e24 (2017).

45. M. Percharde, F. Lavial, J.-H. Ng, V. Kumar, R. A. Tomaz, N. Martin, J.-C. Yeo, J. Gil, S. Prabhakar, H.-H. Ng, M. G. Parker, V. Azuara, Ncoa3 functions as an essential Esrrb

coactivator to sustain embryonic stem cell self-renewal and reprogramming. *Genes Dev.* **26**, 2286–2298 (2012).

46. H. Niwa, K. Ogawa, D. Shimosato, K. Adachi, A parallel circuit of LIF signalling pathways maintains pluripotency of mouse ES cells. *Nature* **460**, 118–122 (2009).

47. M. Wang, L. Tang, D. Liu, Q.-L. Ying, S. Ye, The transcription factor Gbx2 induces expression of Kruppel-like factor 4 to maintain and induce naïve pluripotency of embryonic stem cells. *J. Biol. Chem.* **292**, 17121–17128 (2017).

48. M. A. Blanco, D. B. Sykes, L. Gu, M. Wu, R. Petroni, R. Karnik, M. Wawer, J. Rico, H. Li, W. D. Jacobus, A. Jambhekar, S. Cheloufi, A. Meissner, K. Hochedlinger, D. T. Scadden, Y. Shi, Chromatin-state barriers enforce an irreversible mammalian cell fate decision. *Cell Rep.* **37**, 109967 (2021).

49. S. Ma, B. Zhang, L. M. LaFave, A. S. Earl, Z. Chiang, Y. Hu, J. Ding, A. Brack, V. K. Kartha, T. Tay, T. Law, C. Lareau, Y.-C. Hsu, A. Regev, J. D. Buenrostro, Chromatin potential identified by shared single-cell profiling of RNA and chromatin. *Cell* **183**, 1103–1116.e20 (2020).

50. L. Voortman, C. Anderson, E. Urban, R. Yuan, S. Tran, A. Neuhaus-Follini, J. Derrick, T. Gregor, R. J. Johnston Jr., Temporally dynamic antagonism between transcription and chromatin compaction controls stochastic photoreceptor specification in flies. *Dev. Cell* **57**, 1817–1832.e5 (2022).

51. J.-H. Su, P. Zheng, S. S. Kinrot, B. Bintu, X. Zhuang, Genome-scale imaging of the 3D organization and transcriptional activity of chromatin. *Cell* **182**, 1641–1659.e26 (2020).

52. Y. Takei, J. Yun, S. Zheng, N. Ollikainen, N. Pierson, J. White, S. Shah, J. Thomassie, S. Suo, C.-H. L. Eng, M. Guttman, G.-C. Yuan, L. Cai, Integrated spatial genomics reveals global architecture of single nuclei. *Nature* **590**, 344–350 (2021).

53. W. Zhang, J. Qu, G.-H. Liu, J. C. I. Belmonte, The ageing epigenome and its rejuvenation. *Nat. Rev. Mol. Cell Biol.* **21**, 137–150 (2020).

54. S. Naik, E. Fuchs, Inflammatory memory and tissue adaptation in sickness and in health. *Nature* **607**, 249–255 (2022).

55. S. E. Murphy, A. N. Boettiger, Polycomb repression of Hox genes involves spatial feedback but not domain compaction or phase transition. *Nat. Genet.* **56**, 493–504 (2024).

56. H. D. Ou, S. Phan, T. J. Deerinck, A. Thor, M. H. Ellisman, C. C. O'Shea, ChromEMT: Visualizing 3D chromatin structure and compaction in interphase and mitotic cells. *Science* **357**, eaag0025 (2017).

57. N. Feldman, A. Gerson, J. Fang, E. Li, Y. Zhang, Y. Shinkai, H. Cedar, Y. Bergman, G9a-mediated irreversible epigenetic inactivation of Oct-3/4 during early embryogenesis. *Nat. Cell Biol.* **8**, 188–194 (2006).

58. J. Sinha, J. F. Nickels, A. R. Thurm, C. H. Ludwig, B. N. Archibald, M. M. Hinks, J. Wan, D. Fang, L. Bintu, The H3.3K36M oncohistone disrupts the establishment of epigenetic memory through loss of DNA methylation. *Mol. Cell* **84**, 3899–3915.e7 (2024).

59. L. C. M. Anink-Groenen, T. R. Maarleveld, P. J. Verschure, F. J. Bruggeman, Mechanistic stochastic model of histone modification pattern formation. *Epigenetics Chromatin* **7**, 30 (2014).

60. S. H. Sandholtz, Q. MacPherson, A. J. Spakowitz, Physical modeling of the heritability and maintenance of epigenetic modifications. *Proc. Natl. Acad. Sci. U.S.A.* **117**, 20423–20429 (2020).

61. D. Jost, C. Vaillant, Epigenomics in 3D: importance of long-range spreading and specific interactions in epigenomic maintenance. *Nucleic Acids Res.* **46**, 2252–2264 (2018).

62. D. Michieletto, E. Orlandini, D. Marenduzzo, Polymer model with epigenetic recoloring reveals a pathway for the de novo establishment and 3D organization of chromatin domains. *Phys. Rev. X* **6**, 041047 (2016).

63. W. Ren, H. Fan, S. A. Grimm, Y. Guo, J. J. Kim, J. Yin, L. Li, C. J. Petell, X.-F. Tan, Z.-M. Zhang, J. P. Coan, L. Gao, L. Cai, B. Detrick, B. Çetin, Q. Cui, B. D. Strahl, O. Gozani, Y.

Wang, K. M. Miller, S. E. O'Leary, P. A. Wade, D. J. Patel, G. G. Wang, J. Song, Direct readout of heterochromatic H3K9me3 regulates DNMT1-mediated maintenance DNA methylation. *Proc. Natl. Acad. Sci. U.S.A.* **117**, 18439–18447 (2020).

64. H. Tamaru, E. U. Selker, A histone H3 methyltransferase controls DNA methylation in Neurospora crassa. *Nature* **414**, 277–283 (2001).

65. S. Quenneville, P. Turelli, K. Bojkowska, C. Raclot, S. Offner, A. Kapopoulou, D. Trono, The KRAB-ZFP/KAP1 system contributes to the early embryonic establishment of site-specific DNA methylation patterns maintained during development. *Cell Rep.* **2**, 766–773 (2012).

66. M. K. Y. Seah, Y. Wang, P.-A. Goy, H. M. Loh, W. J. Peh, D. H. P. Low, B. Y. Han, E. Wong, E. L. Leong, G. Wolf, S. Mzoughi, H. Wollmann, T. S. Macfarlan, E. Guccione, D. M. Messerschmidt, The KRAB-zinc-finger protein ZFP708 mediates epigenetic repression at RMER19B retrotransposons. *Development* **146**, dev170266 (2019).

67. H. O'Geen, M. Tomkova, J. A. Combs, E. K. Tilley, D. J. Segal, Determinants of heritable gene silencing for KRAB-dCas9 + DNMT3 and Ezh2-dCas9 + DNMT3 hit-and-run epigenome editing. *Nucleic Acids Res.* **50**, 3239–3253 (2022).

68. J. A. Owen, L. A. Mirny, Chromatin as a three-dimensional memory machine. *Curr. Opin. Struct. Biol.* **95**, 103160 (2025).

69. V. I. P. Keizer, S. Grosse-Holz, M. Woringer, L. Zambon, K. Aizel, M. Bongaerts, F. Delille, L. Kolar-Znika, V. F. Scolari, S. Hoffmann, E. J. Banigan, L. A. Mirny, M. Dahan, D. Fachinetti, A. Coulon, Live-cell micromanipulation of a genomic locus reveals interphase chromatin mechanics. *Science* **377**, 489–495 (2022).

70. J. Tycko, M. V. Van, Aradhana, N. DelRosso, D. Yao, X. Xu, C. Ludwig, K. Spees, K. Liu, G. T. Hess, M. Gu, A. X. Mukund, P. H. Suzuki, R. A. Kamber, L. S. Qi, L. Bintu, M. C. Bassik, Development of compact transcriptional effectors using high-throughput measurements in diverse contexts. bioRxiv 540558 [Preprint] (2023).

71. F. Oceguera-Yanez, S.-I. Kim, T. Matsumoto, G. W. Tan, L. Xiang, T. Hatani, T. Kondo, M. Ikeya, Y. Yoshida, H. Inoue, K. Woltjen, Engineering the AAVS1 locus for consistent and scalable transgene expression in human iPSCs and their differentiated derivatives. *Methods* **101**, 43–55 (2016).

72. H. Xu, I. A. Akinyemi, J. Haley, M. T. McIntosh, S. Bhaduri-McIntosh, ATM, KAP1 and the Epstein-Barr virus polymerase processivity factor direct traffic at the intersection of transcription and replication. *Nucleic Acids Res.* **51**, 11104–11122 (2023).

73. T. Igarashi, M. Mazevet, T. Yasuhara, K. Yano, A. Mochizuki, M. Nishino, T. Yoshida, Y. Yoshida, N. Takamatsu, A. Yoshimi, K. Shiraishi, H. Horinouchi, T. Kohno, R. Hamamoto, J. Adachi, L. Zou, B. Shiotani, An ATR-PrimPol pathway confers tolerance to oncogenic KRAS-induced and heterochromatin-associated replication stress. *Nat. Commun.* **14**, 4991 (2023).

74. U. Schmidt, M. Weigert, C. Broaddus, G. Myers, "Cell detection with star-convex polygons" in *Medical Image Computing and Computer Assisted Intervention – MICCAI 2018* (Springer International Publishing, 2018), pp. 265–273.

75. W. M. Rand, Objective criteria for the evaluation of clustering methods. *J. Am. Stat. Assoc.* **66**, 846–850 (1971).

76. N. C. Durand, J. T. Robinson, M. S. Shamim, I. Machol, J. P. Mesirov, E. S. Lander, E. L. Aiden, Juicebox provides a visualization system for Hi-C contact maps with unlimited zoom. *Cell Syst* **3**, 99–101 (2016).

77. R. Okuta, Y. Unno, D. Nishino, S. Hido, C. Loomis, Cupy: A numpy-compatible library for nvidia gpu calculations, in *Proceedings of Workshop on Machine Learning Systems (LearningSys) in the Thirty-First Annual Conference on Neural Information Processing Systems (NIPS)* (2017), vol. 5.

78. T. Akiba, S. Sano, T. Yanase, T. Ohta, M. Koyama, "Optuna: A next-generation hyperparameter optimization framework" in *Proceedings of the 25th ACM SIGKDD International Conference on Knowledge Discovery & Data Mining* (Association for Computing Machinery, 2019), pp. 2623–2631.

79. D. T. Gillespie, A general method for numerically simulating the stochastic time evolution of coupled chemical reactions. *J. Comput. Phys.* **22**, 403–434 (1976).

80. B. Bintu, L. J. Mateo, J.-H. Su, N. A. Sinnott-Armstrong, M. Parker, S. Kinrot, K. Yamaya, A. N. Boettiger, X. Zhuang, Super-resolution chromatin tracing reveals domains and cooperative interactions in single cells. *Science* **362**, eaau1783 (2018).
